# DSCAM Deficiency Leads to Premature Spine Maturation and Autism-like Behaviors

**DOI:** 10.1523/JNEUROSCI.1003-21.2021

**Published:** 2022-01-26

**Authors:** Peng Chen, Ziyang Liu, Qian Zhang, Dong Lin, Lu Song, Jianghong Liu, Hui-Feng Jiao, Xinsheng Lai, Suqi Zou, Shunqi Wang, Tian Zhou, Bao-Ming Li, Li Zhu, Bing-Xing Pan, Erkang Fei

**Affiliations:** ^1^School of Life Sciences, Nanchang University, Nanchang, 330031, China; ^2^Institute of Life Science, Nanchang University, Nanchang, 330031, China; ^3^School of Basic Medical Sciences, Nanchang University, Nanchang, 330031, China; ^4^State Key Laboratory of Brain and Cognitive Science, Institute of Biophysics, Chinese Academy of Sciences, Beijing, 100101, China; ^5^School of Basic Medical Sciences, Hangzhou Normal University, Hangzhou, 311121, China

**Keywords:** DSCAM, Autism, dendritic spine, NLGN1

## Abstract

Mutations in some cell adhesion molecules (CAMs) cause abnormal synapse formation and maturation, and serve as one of the potential mechanisms of autism spectrum disorders (ASDs). Recently, *DSCAM* (Down syndrome cell adhesion molecule) was found to be a high-risk gene for autism. However, it is still unclear how DSCAM contributes to ASD. Here, we show that DSCAM expression was downregulated following synapse maturation, and that DSCAM deficiency caused accelerated dendritic spine maturation during early postnatal development. Mechanistically, the extracellular domain of DSCAM interacts with neuroligin1 (NLGN1) to block the NLGN1-neurexin1β (NRXN1β) interaction. DSCAM extracellular domain was able to rescue spine overmaturation in *DSCAM* knockdown neurons. Precocious spines in DSCAM-deficient mice showed increased glutamatergic transmission in the developing cortex and induced autism-like behaviors, such as social novelty deficits and repetitive behaviors. Thus, DSCAM might be a repressor that prevents premature spine maturation and excessive glutamatergic transmission, and its deficiency could lead to autism-like behaviors. Our study provides new insight into the potential pathophysiological mechanisms of ASDs.

**SIGNIFICANCE STATEMENT** *DSCAM* is not only associated with Down syndrome but is also a strong autism risk gene based on large-scale sequencing analysis. However, it remains unknown exactly how DSCAM contributes to autism. In mice, either neuron- and astrocyte-specific or pyramidal neuron-specific DSCAM deficiencies resulted in autism-like behaviors and enhanced spatial memory. In addition, DSCAM knockout or knockdown in pyramidal neurons led to increased dendritic spine maturation. Mechanistically, the extracellular domain of DSCAM binds to NLGN1 and inhibits NLGN1-NRXN1β interaction, which can rescue abnormal spine maturation induced by DSCAM deficiency. Our research demonstrates that DSCAM negatively modulates spine maturation, and that DSCAM deficiency leads to excessive spine maturation and autism-like behaviors, thus providing new insight into a potential pathophysiological mechanism of autism.

## Introduction

Proper synapse connections are crucial for the precise assembly of neural circuitry and for normal brain function. Dysregulation of synapse formation and maturation is associated with a variety of neurodevelopmental disorders, including autism spectrum disorders (ASDs) (Forrest et al., 2018). Cell adhesion molecules (CAMs), which are cell surface proteins that mediate cell–cell interactions, play an essential role in neural development, especially in synaptic development and function (Dalva et al., 2007). ASDs manifest with deficits in social interaction and communication, as well as in increased repetitive or restrictive behaviors (Huguet et al., 2013, 2016). However, the etiology underlying ASDs at the molecular, cellular, and system levels remains elusive. ASDs are highly heritable diseases (Bailey et al., 1995; Autism Genome Project Consortium, 2007; Rosenberg et al., 2009). Many genes have been identified as risk factors for ASDs (Bourgeron, 2015). A prominent group among these genes is the set of genes that encode CAMs, such as neuroligins (NLGNs) and neurexin1 (NRXN1). At the cellular and molecular levels, dysfunction of neuronal CAMs is regarded as one of the potential mechanisms of ASDs (J. A. Chen et al., 2015). Mice with mutations of these CAMs display autism-like behaviors and deficits in synapse formation and maturation (Sudhof, 2008). However, it remains unknown how these ASDs-related CAMs impact synapse development. Recently, *DSCAM* (Down syndrome CAM) was found to be one of the genetic risk factors for autism in a large-scale genome-wide association study (Satterstrom et al., 2020). Further research, including whole-genome sequencing (De Rubeis et al., 2014; Yuen et al., 2017), exome sequencing (Iossifov et al., 2014; Ronemus et al., 2014), and targeted sequencing (Stessman et al., 2017), has shown that *DSCAM* is a strong autism risk gene. In some ASD patients, *DSCAM* mutations resulted in premature termination (Iossifov et al., 2014; T. Wang et al., 2016; Stessman et al., 2017; Yuen et al., 2017), suggesting that *DSCAM* loss-of-function variants may cause ASD.

DSCAM is a cell adhesion protein that is highly expressed in the developing nervous system and that plays a vital role in neural development (Yamakawa et al., 1998; Agarwala et al., 2001a, b; Fuerst et al., 2008, 2009). In *Drosophila*, alternative splicing generates thousands of dscam isoforms, which all regulate dendritic self-avoidance and mosaic tiling (Schmucker et al., 2000; Hughes et al., 2007; Matthews et al., 2007; Hattori et al., 2008). Because of the lack of extensive alternative splicing in vertebrates, DSCAM has only two paralogs, DSCAM and DSCAM-like-1 (DSCAML1) (Agarwala et al., 2001b). In vertebrates, DSCAM paralogs play a similar regulatory role in neurite arborization, dendritic self-avoidance, and mosaic tiling (Fuerst et al., 2008, 2009). Moreover, DSCAM plays a critical role in synapse development. In *Drosophila*, dscam was reported to regulate precise synaptic targeting (Millard et al., 2010), and dysregulated dscam levels resulted in altered presynaptic size (Kim et al., 2013; Sterne et al., 2015). Additionally, dscam is involved in mediating *de novo* and learning-related synapse formation in diverse biological models, including chicks and *Aplysia* (Yamagata and Sanes, 2008; H. L. Li et al., 2009). Disruption of glutamatergic transmission and plasticity has been previously documented in DSCAM^2J^-deficient intracortical circuits of the motor cortex, as well as in spinal interneuronal circuits (Thiry et al., 2016; Laflamme et al., 2019). Despite DSCAM's abundance in the brain and its modulation of diverse physiological functions, little is known about how it regulates synapse development in mammals. Therefore, we hypothesized that DSCAM mediates synapse development and that its deficiency can cause autism.

Here, we examined the expression pattern of DSCAM during postnatal synapse formation and maturation, observed the effects of DSCAM deficiency on dendritic spine development, and investigated its potential as an underlying mechanism of ASD. Furthermore, we generated DSCAM-deficient mice to validate whether the deletion of DSCAM in neurons would result in abnormal spine maturation and altered synaptic transmission, as well as autism-like behaviors. These findings describe a novel pathophysiological role of DSCAM in ASDs.

## Materials and Methods

### Animals

Floxed *Dscam* mouse (C57BL/6) was a kind gift from Jane Y. Wu (Institute of Biophysics, Chinese Academy of Science). *GFAP*::Cre transgenic (tg) mouse was purchased from The Jackson Laboratory, and *NEX*-Cre mouse was a kind gift from Yu-Qiang Ding (Institutes of Brain Science, Fudan University). Primers for genotyping are listed in Table 1. Behavior tests were performed with 2- to 3-month male mice. All mice were housed in a constant temperature and humidity chamber at 23°C, and sufficient food and water were administered daily. No more than 5 adult mice per cage were subjected to a 12 h light/dark cycle under standard conditions. All the mice were guaranteed to be hygienic. The animal experiments were conducted following the *Guidelines for the care and use of laboratory animals* promulgated by Nanchang University.

**Table 1.** Primers for mice genotyping

Primers	Sequence (5′→3′)	Size
PP 1	ACCTCCCACAAACAAACCAG	Wt = 396 bp, Floxed = 504 bp
	TGCAGAAGAGTCAAGGCACA	
PP 2	AGACAGGAGGGTACAGAGGA	Wt = 457 bp, Floxed = 565 bp
	GTTGTGACTTTAGGGTTATG	
PP 3	GCTTGCTCATGTGAGCTGGA	Floxed = 420 bp
	GGCTGGACGTAAACTCCTC	
PP 4	TCTGAGGCGGAAAGAACCAG	Floxed = 306 bp
	GCAGCTGAGAGCAAGTCTCACTG	
PP 5	GCTTGCTCATGTGAGCTGGA	Wt = 446 bp
	GCAGCTGAGAGCAAGTCTCACTG	
PP 6 (GFAP-Cre)	ACTCCTTCATAA AGCCCT	Tg = 190 bp
	ATCACTCGTTGCATCGACCG	
PP 7 (NEX-Cre)	GAGTCCTGGAATCAGTCTTTTTC	Cre = 550 bp Wt = 770 bp
	ATCACTCGTTGCATCGACCG	
	CCGCATAACCAGTGAAACAG	

### qRT-PCR analysis

Cortical total RNA was purified from WT mice with Trizol reagent (Invitrogen); 500 ng of total RNA was reverse transcribed to cDNA with oligo (dT) primers. cDNAs were used as the template in qPCR in a 20 μl reaction system containing SYBR GreenER qPCR mix (Invitrogen) with gene-specific primers as shown in Table 2. GAPDH was used as a reference in each sample.

**Table 2.** Gene-specific primers for qPCR

Primers	Sequence (5′→3′)
NLGN1-F	TGATGGGAGTGTCTTGGCAAGC
NLGN1-R	CCGTAGTTTCCTTTGGCAGCCT
NLGN2-F	CGATGTCATGCTCAGCGCAGTA
NLGN2-R	CCACACTACCTCTTCAAAGCGG
NLGN3-F	ATCGGTGCATCCTGTGTCAGTC
NLGN3-R	CACTGGTTGGTAGTTCACAGCC
NLGN4-F	GTGGTGATGACCTACTGGACGA
NLGN4-R	GCAGATAGAGCTGGTCTTTGGG
SALM2-F	GGCATCCGTATGTACCAAGTGC
SALM2-R	GCCAGGTCATTCACTAGGAAGG
SALM4-F	AGGCATCCGCATGTACCAGATC
SALM4-R	TCAGCAGGAAGGAGCGACTGTC
MDGA1-F	ACCAGTGCCTGACCTCAGCATA
MDGA1-R	TTGCCACGAACTTCGCACTGGA
MDGA2-F	CAAGAGAAGCCTTGGTGCAGCT
MDGA2-R	CAGCACTCGTATTGGATAGGCTC
NRXN1-F	ACCGTGCCTTAGCAATCCTTGC
NRXN1-R	GTCGTAGCTCAAAACCGTTGCC
CNTNAP2-F	GTGATGAGACAGGATACAGCGG
CNTNAP2-R	AGTGGTCCACTGCCATCAGGAT
CNTN4-F	CTCCAGCAGAATCCGCACTAAG
CNTN4-R	CTCCATTCTGGTACTCTCGTGAC
CNTN6-F	CTCTGTTGCTGCGGACCTGATT
CNTN6-R	GTTGTCAGGTCCTGGTCTCCAA
DSCAM-F	CATCCGCATGTACGCCAAGAAC
DSCAM-R	GAGATGAGGTGGGTTCCAAGTG
β-catenin-F	GTTCGCCTTCATTATGGACTGCC
β-catenin-R	ATAGCACCCTGTTCCCGCAAAG
N-cadherin-F	CCTCCAGAGTTTACTGCCATGAC
N-cadherin-R	CCACCACTGATTCTGTATGCCG
NrCAM-F	GCAGAGTGAAACATGACCACACC
NrCAM-R	GTGTAGGTTCCGCCATCGTCAT
NCAM-F	GGTTCCGAGATGGTCAGTTGCT
NCAM-R	CAAGGACTCCTGTCCAATACGG
LRRTM2-F	GCCATCGACTTGACAGTGTTCG
LRRTM2-R	AACGGTCGTGAGGGATTTCAGG
LRRTM1-F	CGGCTTGTTCAAGCTCACAGAG
LRRTM1-R	CCACAATGGCAACCTTATTCCGC
SYNCAM-F	GCTTCTGCTGTTGCTCTTCTCC
SYNCAM-R	GACTTGGCAACTGATGGTCGCA
GAPDH-F	CATCACTGCCACCCAGAAGACTG
GAPDH-R	ATGCCAGTGAGCTTCCCGTTCAG
PSD-95-F	TCAGACGGTCACGATCATCGCT
PSD-95-R	GTTGCTTCGCAGAGATGCAGTC
SYN-F	TATGCCACTGCTGAGCCCTTCA
SYN-R	ATGGCAATCTGCTCAAGCATAGC

### Subcellular fractionation

Mouse brain subcellular fractionation was performed as described previously with modifications (Y. N. Wang et al., 2018). The cerebral cortices from adult mice were homogenized in 10 volumes (vol) of HEPES-buffered sucrose (0.32 m sucrose, 4 mm HEPES/NaOH, pH 7.4) with a glass-Teflon homogenizer. The homogenate was centrifuged at 1000 × *g* for 10 min to remove the nuclear fraction and unbroken cells. The supernatant (S1) was then centrifuged at 10,000 × *g* for 15 min to yield the crude synaptosomal fraction (P1) and the supernatant (S2). The P1 pellet was resuspended in 10 vol of HEPES-buffered sucrose and then centrifuged at 10,000 × *g* for another 15 min. The resulting pellet (P2) was lysed by hypo-osmotic shock in water, rapidly adjusted to 4 mm HEPES, and mixed continuously for 30 min (on ice). The lysate was then centrifuged at 25,000 × *g* for 20 min to yield the supernatant (S3, crude synaptic vesicle fraction) and the pellet (P3, lysed synaptosomal membrane fraction). The P3 pellet was resuspended in HEPES-buffered sucrose, carefully layered on top of a discontinuous gradient containing 0.8-1.0-1.2 m sucrose (top to bottom), and centrifuged at 150,000 × *g* for 2 h. Optional step. The S3 was centrifuged at 165,000 × *g* for 2 h to get the synaptic vesicle protein (SV). The sucrose gradient yielded a floating myelin fraction (G1), a light membrane fraction at the 0.8 m/1.0 m sucrose interface (G2), a synaptosomal plasma membrane (SPM) fraction at the 1.0 m/1.2 m sucrose interface (G3), and a mitochondrial fraction as the pellet (G4). The G3 layer was collected, and an equal volume of HEPES-buffered sucrose was added to centrifuge at 20,000 × *g* for 15 min to obtain SPM. The SPM was resuspended with 1% Triton X-100 in 50 mm HEPES/NaOH, pH 8, on ice for 15 min and then centrifuged at 36,000 × *g* for 15 min to yield the supernatant (presynaptic membrane protein [Pre]) and the pellet (postsynaptic density [PSD]), which was solubilized in 2% SDS PBS buffer at room temperature.

### Plasmids and shRNA construction

The constructs expressing full-length mouse DSCAM with a C-terminal His-tag and human DSCAM were purchased from Addgene. To generate the construct expressing secretable extracellular domain (ECD) of DSCAM (FLAG-ECD), mouse DSCAM cDNA encoding 24-1594 amino acids without signal peptide was amplified by PCR and subcloned into PLP2 (new prolactin leader peptide)-FLAG-pCMV6-XL4 downstream of an artificial signal peptide sequence and a FLAG epitope. The constructs of mouse NRXNs (1α-3α and 1β) and NLGNs (1-4) were purchased from Origene. Myc-tagged secretable NRXN1β-ECD was subcloned from full-length NRXN1β. For gene knockdown by RNA interference, pSUPER vector (OligoEngine)-based small hairpin RNAs (shRNAs) of mouse *DSCAM* (sh-DSCAM) and *DSCAM* scramble (sh-control) were constructed. The target sequence of sh-DSCAM was obtained from a previous report: 5′-GTGGGAGAGGAAGTGATAT-3′ (Ly et al., 2008). The sh-control sequence was 5′-AGAGTGGACGTCGATGATTAT-3′. The authenticity of all constructs was verified by DNA sequencing and Western blotting analysis.

### Cell culture and transfection

Human embryonic kidney (HEK) 293 cells and COS-7 cells (African green monkey kidney fibroblast cells) were cultured in DMEM (Invitrogen) supplemented with 10% FBS (Invitrogen). Transient transfection was performed using polyethylenimine (Sigma, 408727), as described previously (P. Chen et al., 2021). Briefly, cells were cultured in 100 mm dishes; and at ∼70% confluence, they were incubated with precipitates formed by 5 μg of plasmid DNA and 280 μl of 0.05% polyethylenimine (w/v). Cells were harvested 24-48 h after transfection.

Cultures of primary hippocampal neurons were prepared from embryonic day (E) 18 Sprague–Dawley rats as described previously (P. Chen et al., 2021). Briefly, hippocampi were isolated and kept separate from one another in HBSS on ice. Following digestion in 0.25% trypsin plus 0.1 mg/ml DNase I (one hippocampus in 1 ml) at 37°C for 20 min, dissociated cells were resuspended in plating media (DMEM supplemented with 10% FBS) and plated at a density of 1 × 10^5^ or 2 × 10^5^ per well onto poly-D-lysine–coated 20 mm coverslips (WHB) in 12-well plates (Corning). Cells were incubated for 4 h before replacing with maintenance medium [neurobasal medium (Invitrogen) supplemented with 2% B-27 supplement (Invitrogen), 1% GlutaMax (Invitrogen), and 1% penicillin/streptomycin (Invitrogen)]. Neurons were maintained at 37°C in 5% CO_2_, with half of the medium changed every 2-3 d. For transfection in neurons, calcium phosphate precipitation was performed as described previously. Briefly, the neurons were serum-starved with preheated DMEM for 2 h at 37°C in 10% CO_2_. For each well of 12-well plate, 1-6 μg DNA in 1-6 μl was mixed with 5 μl 2.5 m CaCl_2_ in ddH_2_O (total volume 50 μl), and further mixed with 50 μl of HEPES-buffered saline containing the following (in mm): 274 NaCl, 10 KCl, 1.4 Na_2_HPO_4_, 15 glucose, and 42 HEPES, pH 7.05. Resulting DNA-calcium phosphate precipitates were added into neurons. Morphology was studied 3-7 d later.

### Cell surface binding assay

For obtaining purified ECD, constructs expressing secretable ECD were transfected into HEK293 cells. After 24 h, the medium was changed to conditional medium (CM) containing 0.5% FBS for another 24 h. Then the CM were collected for immunoprecipitation with the tag antibody and Protein A/G beads. Beads were washed with TBS (50 mm Tris HCl, 150 mm NaCl, pH 8.0) buffer 3 times; then five packed bead volumes of 0.1 m glycine HCl buffer, pH 3.0, were added. Samples were incubated with gentle shaking for 5 min at room temperature. They were then centrifuged at 2500 rpm for 5 min to harvest the supernatant. Afterward, 10 μl of 0.5 m Tris HCl, pH 8.0, with 1.5 m NaCl, was added.

For surface binding assay, HEK293 or COS-7 cells were transfected with indicated constructs. After 24-48 h, purified protein or CM was added to the transfected cells for 1 h incubation at 4°C; then the cells were subjected to immunostaining with indicated antibodies.

### Cell aggregation assay

As described previously (Boucard et al., 2012), HEK293 cells were transfected with indicated constructs, respectively. After 48 h, the cells were trypsinized and the green cells were then mixed with red cells to incubate with gentle agitation at room temperature with DMEM supplemented with 10% FBS, 50 mm HEPES-NaOH, pH 7.4, 10 mm CaCl_2_, and 10 mm MgCl_2_. After 0.5-1 h, the cell mixtures were transferred gently into a 12-well plate and imaged by fluorescence microscopy. The resulting images were analyzed by counting the number of aggregation particles in the field by ImageJ. Cell aggregation particles were defined as four or more clustered cells with at least one red and one green cell.

### Time-lapse imaging and analysis of dendritic spines

Live-imaging of cultured neurons was performed as described previously with modifications (M. Y. Li et al., 2017). Cultured rat hippocampal neurons were transfected by calcium phosphate precipitation at DIV9 and subjected to live-imaging at DIV15. *z*-stack images of secondary dendrites from transfected neurons were imaged every minute for 30 min, using an Olympus FV1000 confocal microscope with a 40 × (NA1.35) objective for time-lapse imaging. Images were collapsed into 2D projections and analyzed with ImageJ software. Stable spines were defined as protrusions with stable morphology during the entire imaging session; newborn spines were those with emerging protrusions after imaging, regardless of the time they emerged and whether they persisted during the entire imaging session; eliminated spines were spines that were present at the beginning of imaging but then disappeared during the imaging session.

### Immunoprecipitation

Immunoprecipitation was performed as described previously (P. Chen et al., 2021). Transfected HEK293 cells were lysed in immunoprecipitation buffer containing the following (in mm): 20 Tris, pH 7.6, 50 NaCl, 1 EDTA, 1 NaF, 0.5% Nonidet P-40 (v/v), with protease and phosphatase inhibitors. Samples were centrifuged at 12,000 × *g* for 20 min at 4°C to remove debris. Lysates (1-2 mg) or CM were incubated with corresponding antibody (1-2 µg) at 4°C for either 3-4 h or overnight and then incubated with 10-15 µl Protein A/G magnetic agarose beads (Pierce) at 4°C for 1 h. Samples were washed with immunoprecipitation buffer and resuspended in SDS sample buffer. Then the samples were subjected to Western blotting.

### Western blotting

For protein expression detection, tissues were homogenized in PBS plus protease and phosphatase inhibitors. Then the homogenates were lysed in an equal volume of 2× RIPA buffer [0.2% SDS (w/v), 1% sodium deoxycholate (w/v), and 2% Nonidet P-40 (v/v) in PBS] plus protease and phosphatase inhibitors. Lysates were centrifuged at 12,000 × g for 20 min at 4°C to remove debris. The supernatants were subjected to Bradford assay (Pierce) to measure protein concentration and diluted in SDS sample buffer. Protein samples (10-20 µg) were resolved by SDS-PAGE and transferred to PVDF membrane (Millipore). The membrane was immunoblotted with primary and secondary antibodies, and immunoreactive bands were visualized by enhanced chemiluminescence under the gel documentation system (Bio-Rad). Densitometric quantification of protein band intensity was performed using ImageJ. Antibodies were diluted with primary antibody dilution buffer (TBS + 1% Triton X-100 + 5% BSA) for Western blotting. Antibodies used in this manuscript are shown in Table 3.

**Table 3.** Antibodies

Target protein	Host species	Source and catalog #	Dilution
DSCAM	Goat	Millipore (AF3666)	1:500
PSD-95	Mouse	Millipore (MAB1598)	1:1000
β-Actin	Rabbit	Millipore (MABT523)	1:2000
FLAG	Mouse	Sigma (1804)	1:2000
NeuN	Mouse	Millipore (MAB377)	1:1000
GFP	Mouse	Santa Cruz Biotechnology (SC-9996)	1:1000
PV	Mouse	Swant (235)	1:10,000
His	Rabbit	Santa Cruz Biotechnology (SC-8036)	1:1000
myc	Mouse	Santa Cruz Biotechnology (SC-40)	1:500
NLGN1	Mouse	NeuroMab (75-160)	1:500
NLGN1	Sheep	Thermo Fisher Scientific (PA5-48050)	1:500
Synaptophysin	Rabbit	ProteinTech (17785-1-AP)	1:1000
Tublin	Rabbit	Santa Cruz Biotechnology (sc-23948)	1:2000
Synapsin I	Mouse	Cell Signaling (D12G5)	1:200

### Immunostaining

Immunostaining of cultured neurons was performed as described previously with modifications (P. Chen et al., 2021). Primary neurons were fixed with 4% PFA/4% sucrose (w/v) for 15 min. After washing 3 times with PBS, neurons were incubated with primary antibody diluted in GDB buffer (30 mm PB, pH 7.4, containing 0.2% gelatin, 0.6% Triton X-100, and 0.9 m NaCl) at 4°C overnight. After washing 3 times with washing buffer (20 mm PB and 0.5 m NaCl), neurons were incubated with the corresponding AlexaFluor-conjugated secondary antibodies (diluted in GDB buffer) at room temperature for 1 h. The images were obtained by FSX100 (Olympus). Immunostaining of brain slices was performed as described previously (Y. N. Wang et al., 2018). Mice were deeply anesthetized with isoflurane and perfused with PBS followed by 4% PFA. Brains were postfixed in 4% PFA at 4°C overnight and dehydrated using 30% sucrose at 4°C for 2 d. Brain samples were rapidly frozen in OCT, cut into 40 μm sections, and mounted on Super Frost Plus slides (Thermo Fisher Scientific). Sections were blocked and permeabilized in PBS containing 0.5% Triton X-100 and 5% goat serum for 2 h at room temperature. Sections were incubated at 4°C overnight with primary antibodies in PBS containing 5% goat serum and 2% BSA. Sections were washed with PBS and then incubated at room temperature for 1-2 h with secondary antibodies. The images were obtained by FSX100 (Olympus).

### Golgi staining

Golgi staining was performed using the FD Rapid GolgiStain Kit following the manufacturer's protocol (FD Rapid GolgiStain Kit, catalog #PK401). Brain tissues were incubated in mixed Solutions A and B for 2 weeks in the dark at room temperature and Solution C for 3 d. Tissues were cut into slices with 80-100 μm thickness, stained with Solutions D and E, dehydrated in gradient ethanol, cleared with xylene, and mounted on slides for imaging. Images of neurons in sensory cortex L2/3 were taken and imported into ImageJ with NeuronJ plugin for analysis. Dendrites were reconstructed and analyzed using the ImageJ with Sholl Analysis plugin, with 10 μm incremental increases in concentric circular diameter from the soma. The images were obtained by FSX100 (Olympus).

### Electrophysiological analysis

Brain slices recording was performed as described previously (Y. N. Wang et al., 2018). Male mice at P12, P21, or P42 were anesthetized with isoflurane (RWD Life Science). Brains were quickly removed to ice-cold oxygenated (95% O_2_/5% CO_2_) cutting solution containing the following (in mm): 120 choline chloride, 2.5 KCl, 7 MgCl_2_, 0.5 CaCl_2_, 1.25 NaH_2_PO_4_, 26 NaHCO_3_, and 25 glucose. Lamellar 300 μm slices of the cortex were cut using VT-1000S Vibratome (Leica Microsystems). The slices were recovered in oxygenated ACSF for 30 min at 32°C and maintained at room temperature (25 ± 1°C) for an additional 1 h before recording. The ACSF contained the following (in mm): 124 NaCl, 2.5 KCl, 2 MgSO_4_, 2.5 CaCl_2_, 1.25 NaH_2_PO_4_, 26 NaHCO_3_, and 10 glucose. All solutions were saturated with 95% O_2/_5% CO_2_ (v/v).

Slices were placed in the recording chamber, which was superfused (2 ml/min) with ACSF at 32°C-34°C. Pyramidal neurons from sensory cortex L2/3 were visualized with infrared optics using an upright microscope equipped with a 40× water-immersion lens (FN-S2N, Nikon) and an infrared CCD camera (IR-1000, DAGE-MTI). Pipettes were pulled by a micropipette puller (P-1000; Sutter Instrument) with a resistance of 3-5 mΩ. The recording was performed with the MultiClamp 700B amplifier and 1550A digitizer (Molecular Device). Series resistance was <20 mΩ and monitored throughout the experiments.

For mEPSC recording, pyramidal neurons were held at −70 mV in the presence of 20 μm bicuculline (Tocris Bioscience), with the pipette solution containing the following (in mm): 125 K-gluconate, 5 KCl, 10 HEPES, 0.2 EGTA, 1 MgCl_2_, 4 Mg-ATP, 0.3 Na-GTP, and 10 phosphocreatine (pH 7.35, 290-295 mOsm). mEPSCs were recorded in the presence of 1 μm TTX.

For mIPSC recording, pyramidal neurons were held at −70 mV in the presence of 20 μm CNQX (Sigma) and 100 μm DL-AP5 (Tocris Bioscience), with the pipette solution containing the following (in mm): 130 CsCl, 5 Cs-methanesulfonate, 10 HEPES, 0.2 EGTA, 1 MgCl_2_, 4 Mg-ATP, 0.3 Na-GTP, 10 phosphocreatine, and 5 QX-314 (pH 7.35, 290-295 mOsm). mIPSCs were recorded in the presence of 1 μm TTX and filled with the solution containing the following (in mm): 125 Cs-methanesulfonate, 5 CsCl, 10 HEPES, 0.2 EGTA, 1 MgCl_2_, 4 Mg-ATP, 0.3 Na-GTP, 10 phosphocreatine, and 5 QX314 (pH 7.4, 285 mOsm). All the chemicals were purchased from Sigma.

### Electron microscopy

Electron microscopy studies were performed as described previously with modifications (Yang et al., 2017). Briefly, anesthetized mice were perfused transcardially with PBS and followed by PBS containing 2% glutaraldehyde and 4% PFA. Brains were postfixed at 4°C overnight. Ultrathin sections (70 nm) of the sensory cortex L2/3 region were cut and stained with 2% uranyl acetate (v/v) and Reynolds lead citrate, and they were analyzed with a Hitachi H-7650 transmission electron microscope. Symmetric and asymmetric synapses were visually confirmed and manually counted by investigators unaware of genotypes. Also, synaptic ultrastructural specializations, such as area, thickness, and length of PSD, and presynaptic vesicle numbers were analyzed by investigators blind to genotypes.

### Behavioral analysis

Behavioral analysis was conducted with 8- to 10-week-old male mice by investigators unaware of genotypes. Mice were handled for 3 d, and 10 min for each day for each mouse before behavioral tests.

For open field test (OFT), mice were placed in a chamber (50 × 50 cm). An overhead camera and tracking software (Med Associates) were used to monitor the mouse movement for 10 min. Total distance and time traveled in the center (15 × 15 cm) were measured. The chamber was cleaned with 75% ethanol and wiped with paper towels after each trial. During a 10 min OFT period, the amount of time spent grooming and the circling behavior were measured manually.

Elevated plus maze (EPM) was performed as described previously (Chanques et al., 2018). The platform was elevated 74 cm above the floor. It consisted of two closed arms (35 × 6 × 22 cm), two open arms (35 × 6 cm), and a central zone (6 × 6 cm). Mice were placed on the central zone and faced an open arm. Mice could freely explore the platform for 10 min. The total time spent in the open arms and the entries to open arms were recorded by the monitoring software (Med Associates). The apparatus was cleaned with 75% ethanol after each trial.

Three-chamber social preference test was performed as described previously with minor modifications (Moy et al., 2004). The apparatus was a transparent Plexiglas rectangular box (40 × 72 cm) with three equal transparent partitions (40 × 24 cm), including left, right, and center chambers. Two wire cups were placed in the left and right chambers for each one. First, the experimental mouse was placed in the center and allowed to freely explore the three chambers for 10 min. After that, an age- and gender-matched stranger C57BL/6J mouse (S1) was placed in one of the two wire cages, and on the other side were the empty wire cages (E). Then, the test mouse was placed in the center for another 10 min session. The test mouse would now choose between S1 and E. After that, a second age- and gender-matched C57BL/6J stranger mouse (S2) was placed in another wire cage. Finally, the test mouse was placed in the center for the last 10 min session. Thus, the test mouse would now choose between an already familiar mouse (S1) and a new stranger mouse (S2). The mouse's movement was recorded by a video-tracking system (Med Associates). Time spent in each chamber and sniffing time within a 1 cm distance to each wire cage (interaction time) were measured. Social preference index was calculated as described previously (Chiang et al., 2018): [interaction time (S1) – interaction time (E)]/[interaction time (S1) + interaction time (E)] for each genotype; [interaction time (S2) – interaction time (S1)]/[interaction time (S2) + interaction time (S1)] for each genotype.

The Barnes circular maze was performed as described previously with modifications (Rosenfeld and Ferguson, 2014). The Barnes circular maze is a planar, round, white Plexiglas platform (75 cm diameter), 1 m above the floor, with 18 evenly spaced holes (7 cm diameter). A black escape box was placed under one hole. During the trial, a 500 lux light was turned on. After each trial, the platform and the escape box were cleaned thoroughly with 75% ethanol. The test consists of a 4 d training trial and a probe trial. One day before the training trial, test mice were habituated in the target box for 3 min. The training trial was repeated for 4 consecutive days, 3 times each day with 20 min intervals. At the beginning of the training trial, a test mouse was placed in a cylindrical holding chamber (8 cm diameter) located in the maze center. After 15 s of holding time, the mouse was allowed to search for the target hole for 3 min. If the mouse failed to find the target hole in 3 min, it was gently guided into the target hole by the investigator's hands. When the mouse entered the escape box, the light was turned off and the mouse remained undisturbed for 1 min. The mouse's movement was recorded, and the number of errors and the latency to find the target hole were measured during the training trials by video tracking software. On day 5, the probe trial was performed with each mouse. The escape box was removed, and the test mouse was allowed to find the target hole freely for 90 s. During the probe trial, the total distance traveled and the latency to find the target hole were measured; percent correct pokes and percent time in the target area were also measured.

All behavioral results were analyzed by investigators blind to genotypes.

### Statistical analysis

Statistical analysis was done by the GraphPad Prism version 7.0 (GraphPad Software). Before being analyzed, all data in our study were checked by the D'Agostino–Pearson omnibus normality test to prove they came from a Gaussian distribution. Two-way ANOVA that analyzes more than two parameters was used in behavioral and body weight studies. All statistical analyses were presented as mean ± SEM and analyzed by two-tailed Student's *t* test or one-way ANOVA, including behavioral tests, morphologic analysis, electrophysiological studies, and Western blotting. For the data that did not conform to the Gaussian distribution, we used Mann–Whitney *U* test or Kruskal–Wallis test for the statistical analysis. Otherwise, values of *p* < 0.05 were considered statistically significant.

## Results

### DSCAM expression is downregulated during synapse maturation

**Figure 1.**
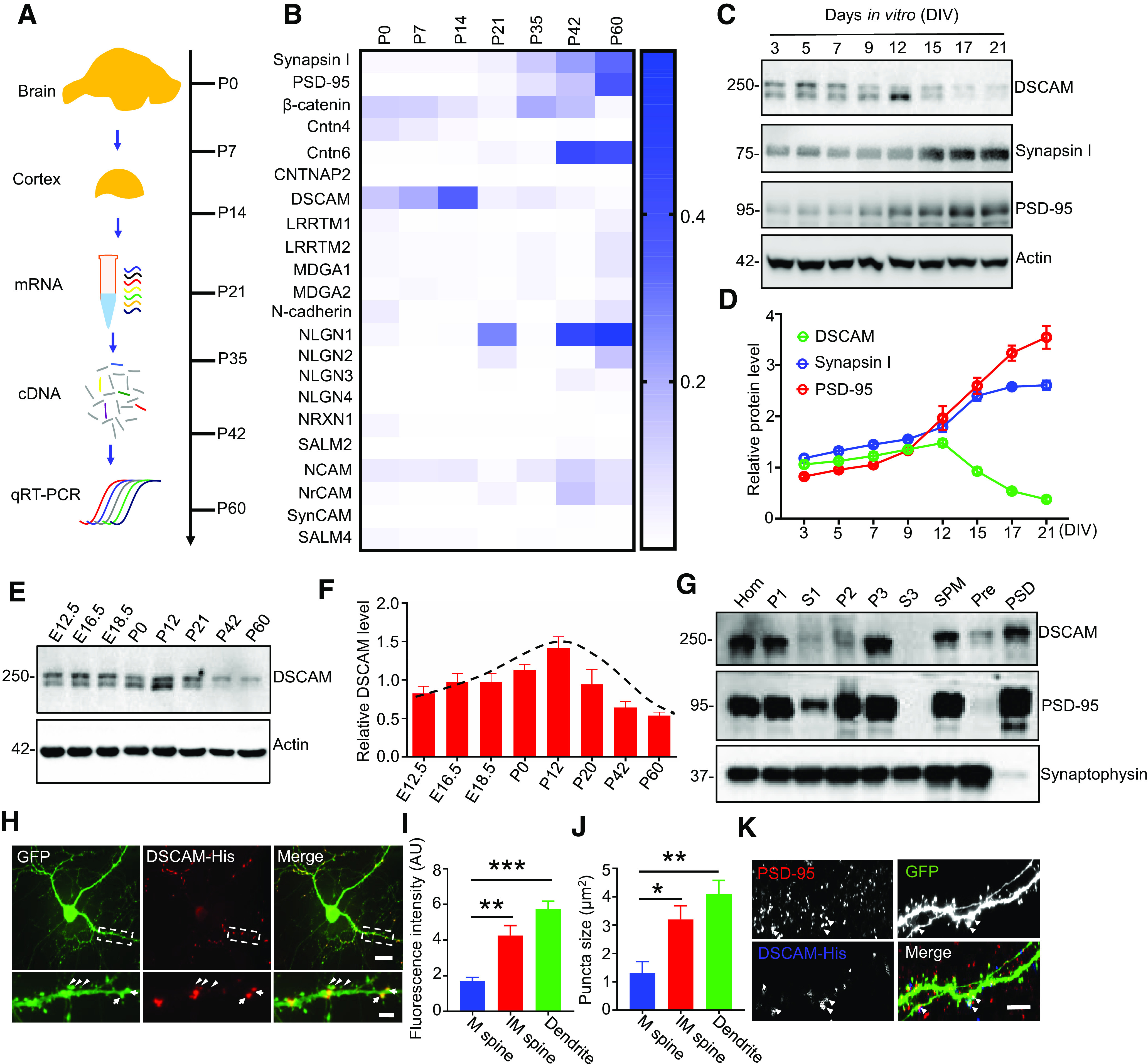
Downregulated expression of DSCAM during synapse maturation. ***A***, ***B***, The mRNA levels of selected CAMs during postnatal synapse development. WT mice cortex was isolated from P0 to P60 for mRNA extraction and qRT-PCR analysis (***A***). Hot map analysis of qRT-PCR results for selected CAMs (***B***). *N* = 3 mice for each age. ***C***, ***D***, DSCAM protein level in cortical neurons from DIV3 to DIV21. Primary cortical neurons were lysed at indicated DIV for Western blotting (***C***). Quantitative analysis of relative protein levels in ***C***; the relative protein levels of all three proteins were set to 1 (***D***). Data were from three independent experiments and shown as mean ± SEM. ***E***, ***F***, DSCAM expression during brain development. Whole-brain homogenates from WT mice at different (indicated) stages from E12.5 to P60 were subjected to Western blotting with the anti-DSCAM antibody (***E***). Quantitative analysis of data in ***E*** (***F***). *N* = 3 mice for each age. ***G***, Distribution of DSCAM in PSD fraction. Subcellular fractions of the WT brains were blotted with anti-DSCAM, anti-PSD-95 (a PSD marker), and anti-synaptophysin (a presynaptic marker). S1, Supernatant 1; P1, pelleted nuclear fraction; S2, supernatant 2; P2, washed crude synaptosomal fraction; S3, crude synaptic vesicle fraction; SV, synaptic vesicle fraction; P3, lysed synaptosomal membrane fraction; Pre, presynaptic fraction. ***H-K***, Distribution of DSCAM in neurons. Representative images of DSCAM-His distribution in dendrites (***H***) and in PSD (***K***). Cortical neurons were isolated from P0 pups, cotransfected with DSCAM-His and GFP at DIV9, and fixed for staining with anti-His and anti-PSD-95 antibodies at DIV17. ***H***, Arrow indicates mature (M) dendritic spine. Arrowhead indicates immature spine (IM) dendritic spine. Scale bars: top, 32 μm; bottom, 10 μm. Quantitative analysis of DSCAM-His fluorescence intensity (***I***) and puncta size (***J***) in ***H***. *N* = 11 neurons. *p < 0.05; **p < 0.01; ***p < 0.001; one-way ANOVA. ***K***, Arrowheads indicate DSCAM-His that was colocalized with PSD-95. Scale bar, 10 μm.

CAMs are critical for synapse formation and maturation, and their expression is regulated during this developmental stage (Foldy et al., 2016). To characterize the expression patterns of CAMs during synapse development, we performed qRT-PCR with mRNA extracted from mouse cortex on postnatal days 0 (P0) to 60 (P60) (Fig. 1*A*). As shown in Figure 1*B*, the expression of most CAMs, such as NLGNs, increased gradually during this stage, consistent with synapsin I and PSD-95 expression patterns. Intriguingly, unlike synapsin I and PSD-95, the expression of DSCAM increased from P0 to P14, but dramatically decreased afterward (Fig. 1*B*). When investigated *in vitro*, DSCAM protein levels in primary cortical neurons similarly increased from DIV3 to DIV12; this phenomenon was also followed by a decrease in DSCAM protein levels (Fig. 1*C*,*D*). Synapse formation commences in the first postnatal week and peaks at P14, and synapse maturation continues thereafter (Farhy-Tselnicker and Allen, 2018). In parallel, the expression of DSCAM in the brain gradually increased from embryonic day 12.5 (E12.5) until it reached its peak at P12, and then decreased until P60 (Fig. 1*E*,*F*). This expression pattern of DSCAM suggests that its level is downregulated during synapse maturation compared with other CAMs. We also examined the subcellular distribution of DSCAM in mouse brains. PSD fractionation assays revealed that DSCAM was localized to the PSD area (Fig. 1*G*). To confirm the distribution of DSCAM in neurons, we cotransfected His-tagged full-length DSCAM plus GFP into cultured neurons at DIV9 and stained them with anti-His antibody at DIV17. As shown in Figure 1*H*, *J*, DSCAM was mainly distributed and clustered in GFP-labeled dendrites and dendritic spines. Dendritic spines are morphologically divided into mature and immature spines. Mature spines are mushroom-like, with a width of spine head to neck ratio ≥ 1.5; immature spines have a spine head to neck ratio < 1.5. Interestingly, the fluorescence intensity and size of DSCAM-His puncta were markedly increased in dendritic shafts and in immature spines, compared with those in mature spines. Furthermore, costaining with PSD-95 demonstrated the colocalization of DSCAM-His with PSD-95 (Fig. 1*K*). Together, these results provide an indication of DSCAM functions in spine maturation.

### DSCAM deficiency results in increased spine maturation in the sensory cortex

**Figure 2.**
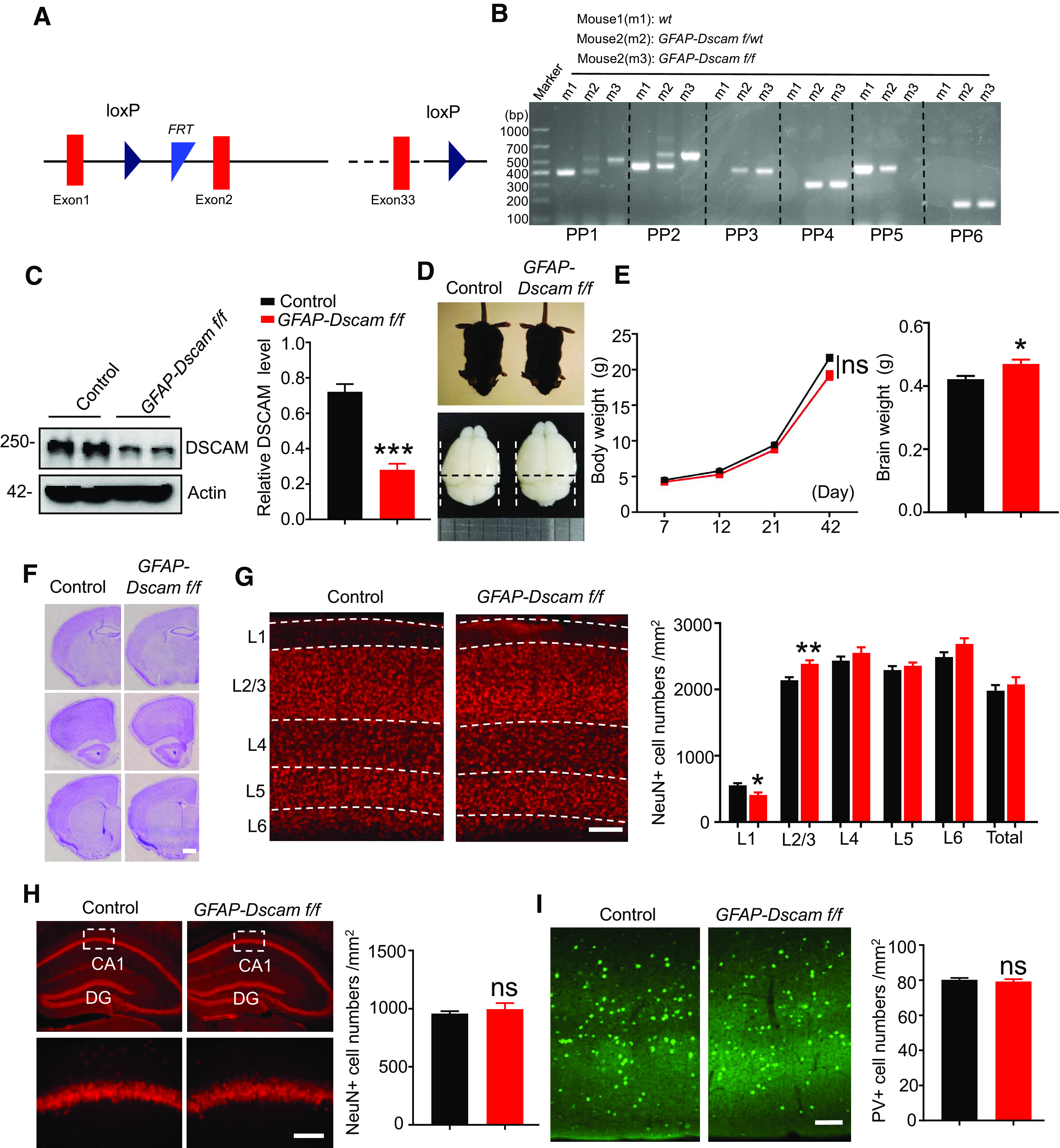
Normal gross brain structure of *GFAP-Dscam f/f*. ***A***, The genomic structure of floxed *Dscam* mice. The first loxp (first loxP) was inserted between exon1 and exon2, and the second loxp (second loxP) was inserted after the last exon (exon33). ***B***, Genotyping of WT (*wt*, m1), *GFAP-Dscam f/wt* (m2), and *GFAP-Dscam f/f* (m3) mice. Primer pair 1 (PP1) and PP2 were for first loxP, PP3 and PP4 were for second loxP, PP5 was for wt allele, and PP6 was for *GFAP*-Cre transgene. ***C***, DSCAM expression is decreased in the brain of *GFAP-Dscam f/f* mice. Whole-brain lysates from *GFAP-Dscam f/f* and control mice were probed with anti-DSCAM antibody. Actin served as a loading control. The relative DSCAM level (band intensity of DSCAM/actin) was quantified. *N* = 4 mice for control, *n* = 5 mice for *GFAP-Dscam f/f* mice. ***p < 0.001 (Student's *t* test). Data are mean ± SEM. ***D-F***, *GFAP-Dscam f/f* mice displayed normal body weight and gross brain structure, but increased brain weight. Representative appearance and brain images of *GFAP-Dscam f/f* and control mice (***D***). Quantitative analysis of body and brain weight for each genotype (***E***). Brain weight: *n* = 5 for control, *n* = 6 for *GFAP-Dscam f/f* mice. Data are mean ± SEM. NS, *p* > 0.05. *p < 0.05 (Student's *t* test). Representative Nissl-staining images of brains from each genotype. Scale bar: ***F***, 1 mm. ***G-I***, *GFAP-Dscam f/f* mice displayed normal densities of NeuN^+^ and PV^+^ neurons. Representative NeuN immunostaining images and quantitative analysis of NeuN^+^ cell densities in sensory cortex (***G***) and hippocampus (***H***) of each genotype. Scale bars: ***G***, 100 μm; ***H***, 32 μm. *N* = 32 slices from 4 control mice, *n* = 43 slices from 5 *GFAP-Dscam f/f* mice. Data are mean ± SEM. NS, *p* > 0.05 (Student's *t* test). Representative PV immunostaining images and quantitative analysis of PV^+^ cell densities in the sensory cortex of each genotype (***I***). Scale bar, 100 μm. *N* = 17 slices from 4 control mice, *n* = 21 slices from 5 *GFAP-Dscam f/f* mice. Data are mean ± SEM. NS, *p* > 0.05, Student's *t* test.

As DSCAM expression was downregulated during the spine maturation period, we were curious as to whether DSCAM deficiency would affect spine density and/or maturation *in vivo*. A previous study reported that *Dscam*-null C57BL/6 mice died within 24 h of birth (Amano et al., 2009), so we generated *Dscam* floxed mice (*Dscam f/f*) (Fig. 2*A*,*B*) and crossed them with a *hGFAP::Cre* line, where Cre is expressed in neural progenitor cells at E13.5 and in all forebrain neurons and astrocytes of mice (Zhuo et al., 2001). Compared with that of control mice (*Dscam f/f*), the expression of DSCAM in the brain was abated by ∼60% in resulting *hGFAP::Cre*; *Dscam f/f* (hereafter referred to as *GFAP-Dscam f/f*) mice (Fig. 2*C*), in which DSCAM was ablated in neurons and astrocytes. Next, we examined whether DSCAM deficiency could alter neural development. As shown in Figure 2*D–F*, *GFAP-Dscam f/f* mice, while displaying a similar body weight to that of control mice and showing no global morphologic deficits, ultimately displayed increased brain weight. The density of NeuN^+^ neurons was decreased in layer 1 (L1) and increased in L2/3 in the cortex of *GFAP-Dscam f/f* mice (Fig. 2*G*). However, the total number of NeuN^+^ neurons in the cortex (Fig. 2*G*) and hippocampus (Fig. 2*H*) remained within normal ranges. The number of PV^+^ interneurons in the cortex was also unchanged (Fig. 2*I*).

**Figure 3.**
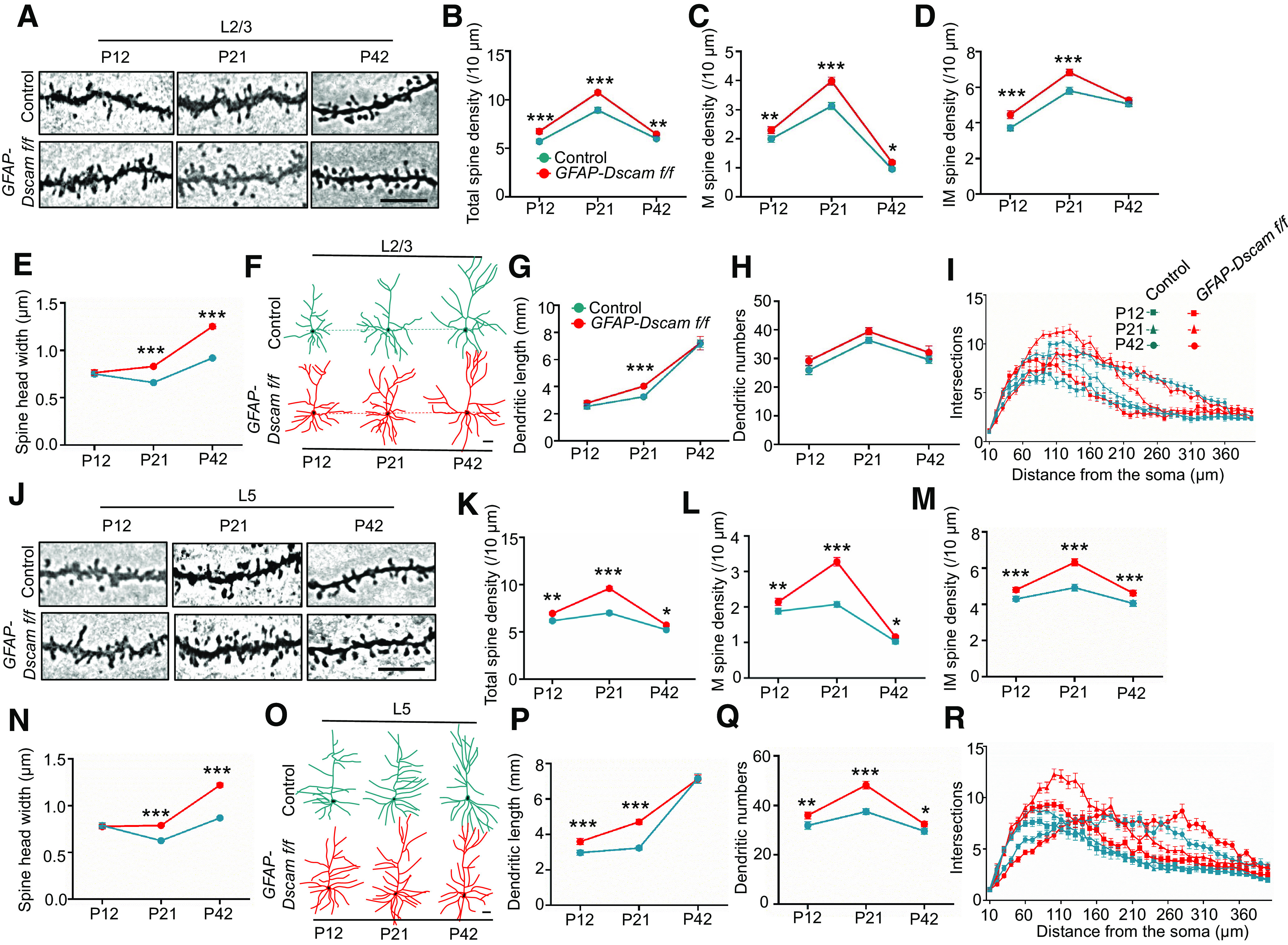
Abnormal spine maturation in sensory cortex of *GFAP-Dscam f/f* mice. ***A-R***, *GFAP-Dscam f/f* mice displayed increased spine maturation in the sensory cortex (***A-E***,***J-N***) and increased dendritic arborization at P21 (***F-I***,***O-R***). Representative Golgi staining images of dendritic spines in sensory cortex from P12, P21, and P42 male mice of each genotype (***A***,***J***). Scale bar, 10 μm. Quantitative analysis of total (***B***,***K***), M (***C***,***L***), and IM (***D***,***M***) spine density, as well as spine head width (***E***,***N***). Representative dendrite trace images from Golgi staining in sensory cortex L2/3 (***F***) and L5 (***O***) of each genotype at P12, P21, and P42. Scale bars, 32 μm. Quantitative analysis of dendritic length (***G***,***P***), branches (***H***,***Q***), and complexity (***I***,***R***) in ***F*** and ***O***. *N* = 5 mice for each genotype. Data are mean ± SEM. NS, *p* > 0.05. *p < 0.05; **p < 0.01; ***p < 0.001; two-way ANOVA.

Neurons in the sensory cortex L2/3 receive multiple inputs and are highly plastic; they are involved in context-specific sensory information processing and in controlling the gain of cortical output (Feldman and Brecht, 2005; Feldman, 2009; Petersen and Crochet, 2013). We used Golgi staining to examine dendritic spine development from P12 to P42 in sensory cortex L2/3 of *GFAP-Dscam f/f* mice. As shown in Figure 3*A–D*, the densities of both total and mature spines were greater in *GFAP-Dscam f/f* mice than in control mice from P12 to P42, while the number of immature spines was greater at P12 and P21, but not P42. Surprisingly, the width of spine heads was also greater at P21 and P42 (Fig. 3*E*) in *GFAP-Dscam f/f* mice. Similar results were observed in sensory cortex L5 (Fig. 3*J–N*). As with a previous study (Maynard and Stein, 2012), the dendritic lengths and branches in sensory cortex L2/3 (Fig. 3*F–H*) and L5 (Fig. 3*O–Q*) were increased in *GFAP-Dscam f/f* mice. Dendritic complexity was increased in *GFAP-Dscam f/f* mice at P21, but it returned to normal levels by P42 (Fig. 3*I*,*R*). These results suggest that DSCAM deficiency in forebrain neurons and astrocytes increases mature spine densities and sizes in the sensory cortex.

**Figure 4.**
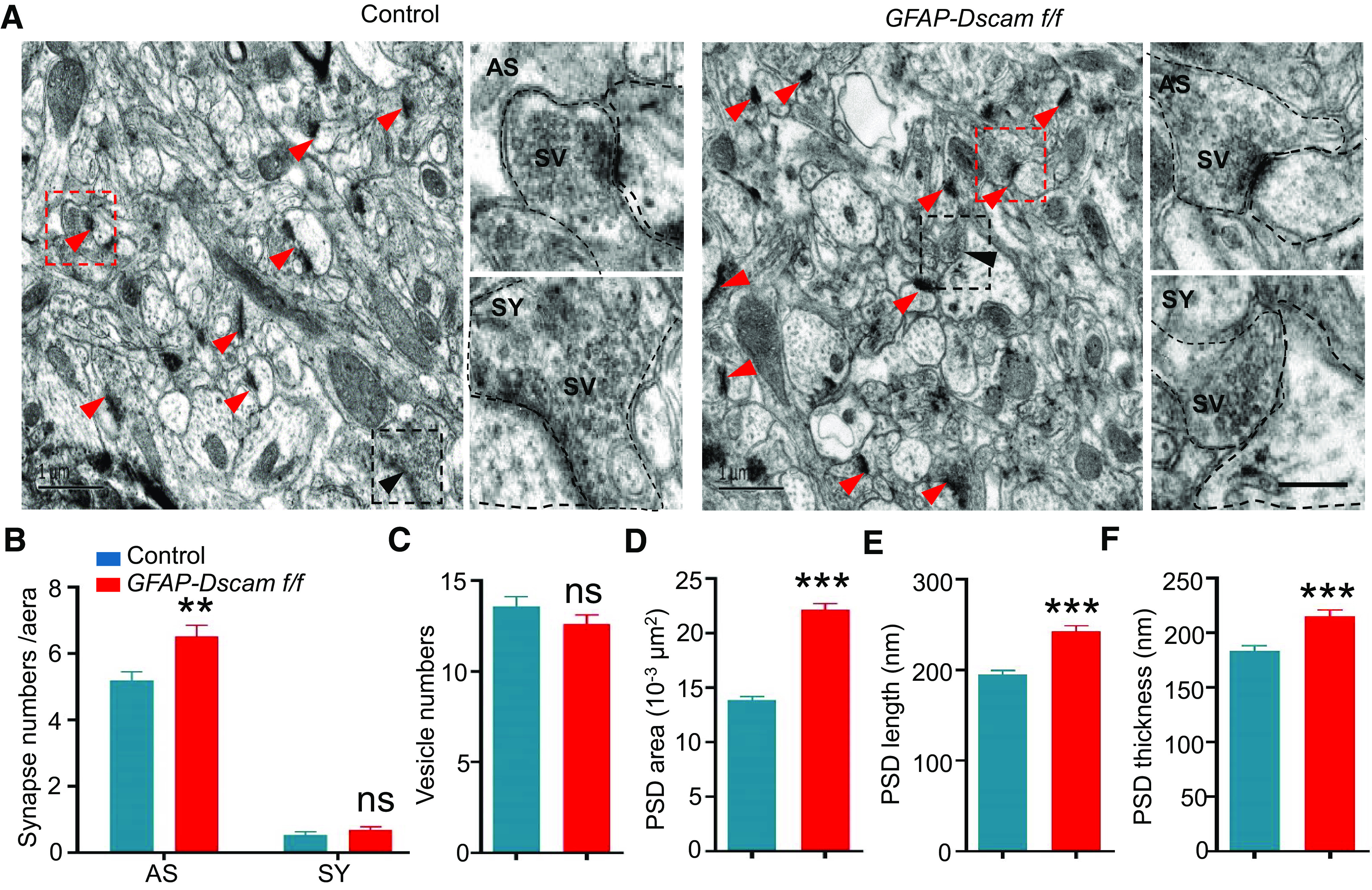
Increased asymmetric synapse number and PSD size in sensory cortex of *GFAP-Dscam f/f* mice. ***A-F***, *GFAP-Dscam f/f* mice displayed increased density of asymmetric (AS) synapses and enlarged PSD area. Representative electron microscopy images of sensory cortex L2/3 from each genotype (***A***). For each genotype: Scale bars: left enlarged insets, 1 μm; right enlarged insets, 0.5 μm. Red arrowheads indicate AS synapses Black arrowheads indicate symmetric (SY) synapses. Red box represents enlarged insets of AS synapses. Black box represents enlarged insets of SY synapses. ***B-F***, Quantitative analysis of data in ***A***. *N* = 3 for each genotype. Data are mean ± SEM. **p < 0.01; ***p < 0.001; Student's *t* test, two-way ANOVA.

The size or volume of spine heads is strongly correlated with the PSD area (Harris et al., 1992; Arellano et al., 2007). A larger spine volume indicates a larger PSD area and a stronger synaptic strength (Holtmaat and Svoboda, 2009). To confirm this relationship, we performed electron microscopy analysis and found that the number of asymmetric (excitatory) synapses was increased in sensory cortex L2/3 in *GFAP-Dscam f/f* mice, while the number of symmetric (inhibitory) synapses was unchanged (Fig. 4*A*,*B*). The presynaptic vesicle number was similar in control and *GFAP-Dscam f/f* mice (Fig. 4*C*). However, the area, length, and thickness of the PSD were all greater in *GFAP-Dscam f/f* mice (Fig. 4*D–F*). Together, these results indicate that DSCAM deficiency increases spine maturation in the sensory cortex.

### Glutamatergic transmission is elevated in the L2/3 sensory cortex of DSCAM-deficient mice

**Figure 5.**
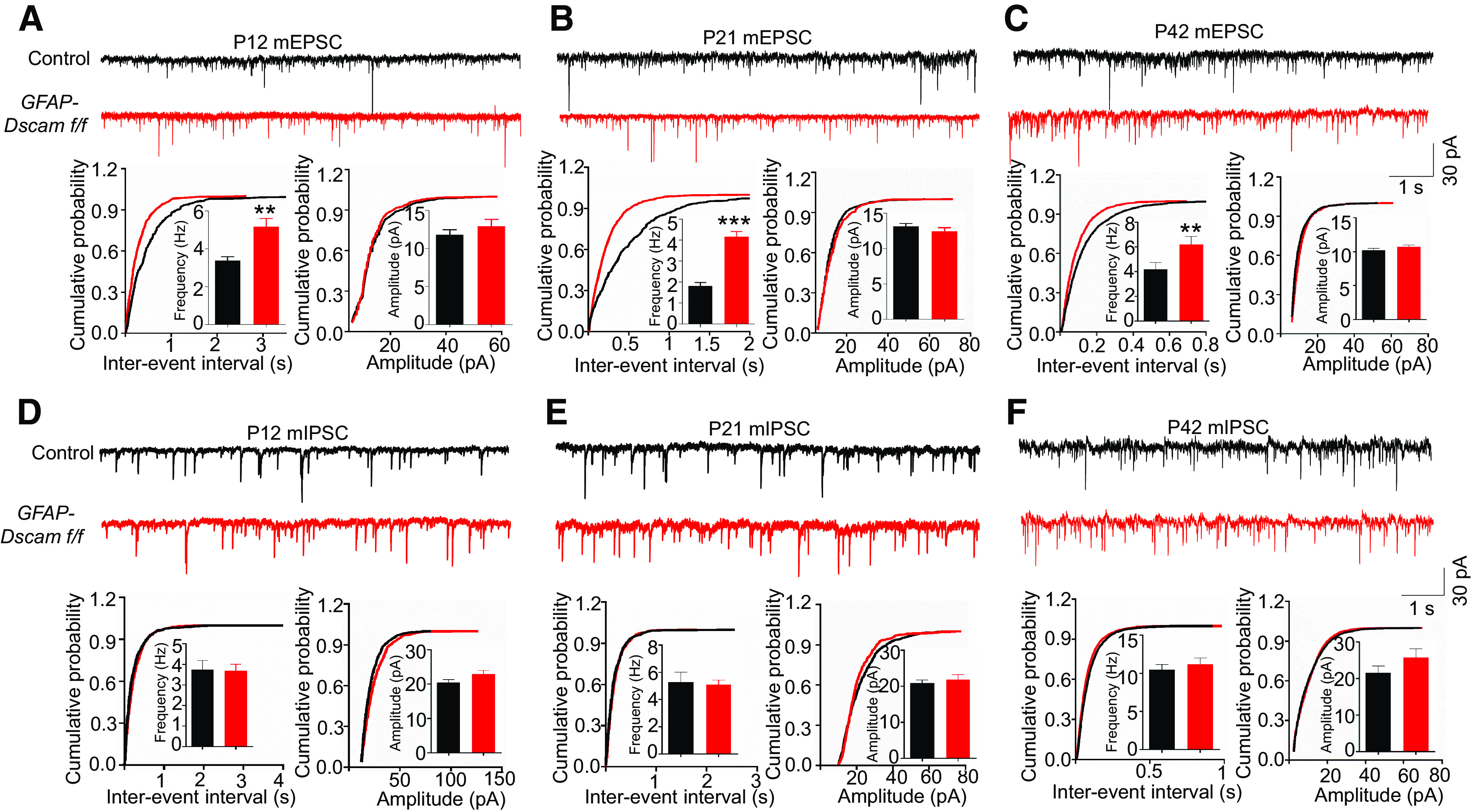
Increased glutamatergic transmission in sensory cortex L2/3 of *GFAP-Dscam f/f* mice. ***A-F***, *GFAP-Dscam f/f* mice displayed increased mEPSC frequency, but normal mIPSC. The sensory cortical slices of each genotype at P12 (***A***,***D***), P21 (***D***,***E***), and P42 (***C***,***F***) were collected for whole-cell voltage-clamp recording of mEPSCs (***A-C***) and mIPSCs (***D-F***). Top panels, Representative traces of mEPSCs (***A-C***) and mIPSCs (***D-F***). Calibration: 30 pA, 1 s. Bottom panels, Histogram summary and cumulative probability plots of mEPSC (***A-C***) and mIPSC (***D-F***) interevent intervals and amplitude. P12: *n* = 34 neurons from 4 control, *n* = 16 neurons from 5 *GFAP-Dscam f/f* mice. P21: *n* = 20 neurons from 5 control, *n* = 22 neurons from 5 *GFAP-Dscam f/f* mice. P42: *n* = 17 neurons from 4 control, *n* = 17 neuron from 4 *GFAP-Dscam f/f* mice. Data are mean ± SEM. **p < 0.01; ***p < 0.001; Student's *t* test.

Dendritic spine density and morphology are critical for synaptic transmission strength and synaptic stability (Alvarez and Sabatini, 2007; Yuste, 2013). An increase in the number of matured spines corresponds to an increase in glutamatergic transmission. We examined whether DSCAM deficiency alters neurotransmission in the cortex during development by recording mEPSCs and mIPSCs in sensory cortex L2/3 of *GFAP-Dscam f/f* mice at P12, P21, and P42. Notably, *GFAP-Dscam f/f* mice displayed an increase in mEPSC frequency at all three time points, but mEPSC amplitudes were not changed (Fig. 5*A–C*). Neither mIPSC frequencies nor amplitudes were altered in *GFAP-Dscam f/f* mice (Fig. 5*D–F*). Together, these results suggest that DSCAM deficiency increases glutamatergic transmission in sensory cortex L2/3.

### Knockdown of DSCAM in primary neurons disrupts spine dynamics and maturation

**Figure 6.**
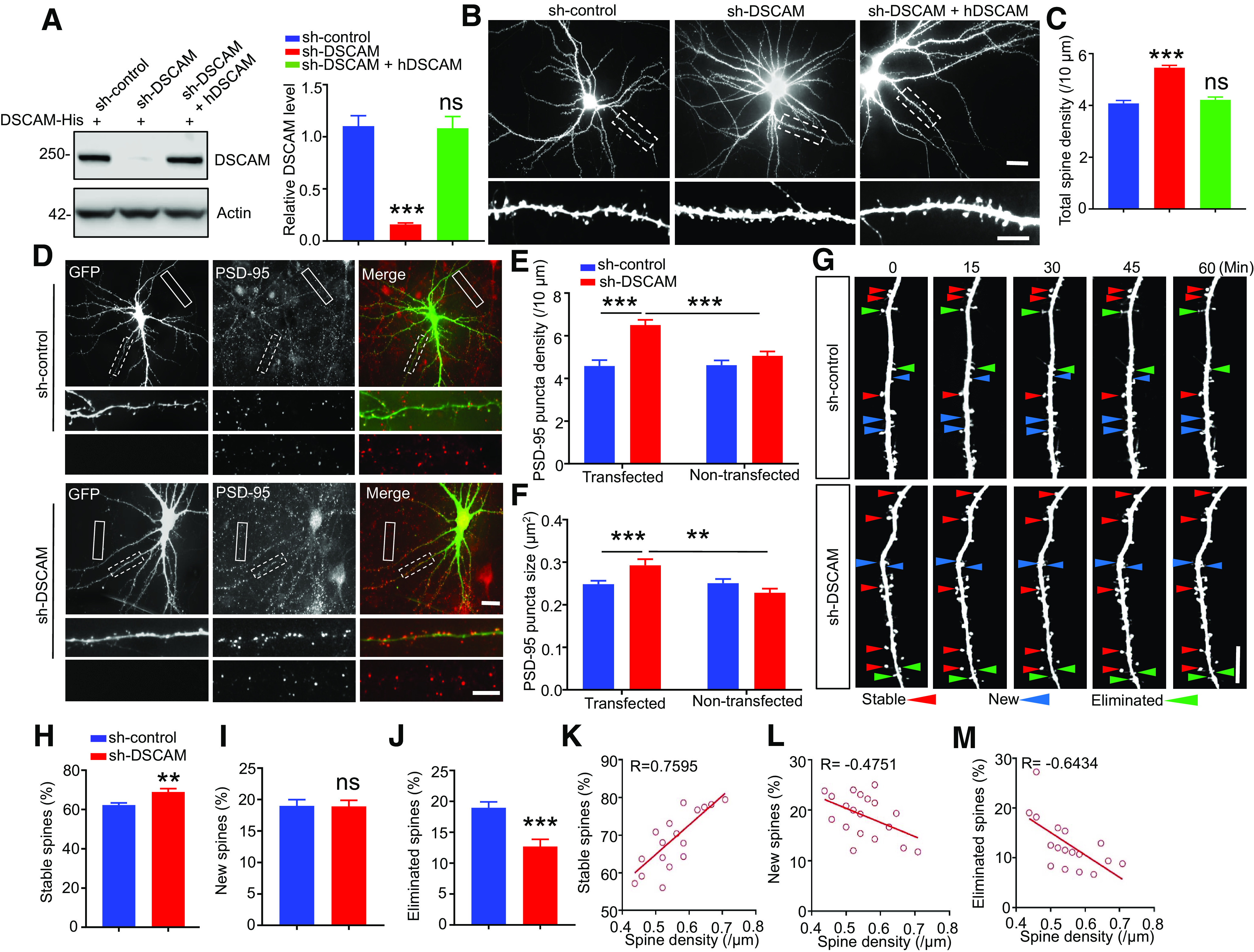
Increased spine maturation and stability in DSCAM knockdown neurons. ***A***, Knockdown of overexpressed mouse DSCAM-His in HEK293T cells. HEK293T cells were cotransfected with mouse DSCAM-His, small hairpin RNA (sh-RNA) for mouse/rat DSCAM (sh-DSCAM) or control scramble sh-RNA (sh-control), and human DSCAM (hDSCAM). Twenty-four hours after transfection, cells were lysed and subjected to Western blotting with anti-DSCAM antibody. Data were from three independent experiments and are shown as mean ± SEM. NS, *p* > 0.05. ***p < 0.001 (Student's *t* test). ***B***, ***C***, Knockdown of DSCAM in cultured cortical neurons increased mature spine density. Representative images of pyramidal neuron morphology and spine density in cortical neurons (***B***). Neurons were isolated from E18 rat to culture and transfected with GFP plus sh-DSCAM or sh-control and hDSCAM or not at DIV9, and they were fixed for staining at DIV17. Scale bars: top, 16 μm; bottom, 10 μm. Statistical analysis of total spine density in ***B*** (***C***). *N* = 29 neurons for sh-control, *n* = 79 neurons for sh-DSCAM, *n* = 22 neurons for shDSCAM + hDSCAM. NS, *p* > 0.05. ***p < 0.001 (Student's *t* test). Data are mean ± SEM. ***D-F***, Knockdown of DSCAM in cultured cortical neurons increased the density and size of PSD-95 puncta. Representative images of PSD-95 staining in cortical neurons (***D***). Primary cortical neurons were transfected with sh-DSCAM or sh-control plus GFP at DIV9, and they were fixed for PSD-95 staining at DIV17. Dotted line box represents dendrite of transfected neurons (enlarged in middle panels). Solid line box represents dendrite of nontransfected neurons (enlarged in bottom panels). Scale bars: top, 16 μm; bottom, 10 μm. Quantitative analysis of data in ***D*** for the density (***E***) and size (***F***) of PSD-95 puncta. *N* = 21 neurons for transfected and nontransfected, respectively, in sh-control; *n* = 49 neurons for transfected and nontransfected, respectively, in sh-DSCAM. **p < 0.01; ***p < 0.001; two-way ANOVA. Data are mean ± SEM. ***G***, Representative images of time-lapse imaging from cortical neurons transfected with sh-DSCAM or sh-control taken at 5 adjacent time points during the 60 min live-imaging period. Scale bar, 10 μm. *N* = 21 neurons for sh-control, *n* = 19 neurons for sh-DSCAM. ***H-J***, Quantitative analysis for percentages of stable (***H***), new (***I***), and eliminated spines (***J***). NS, *p* > 0.05. **p < 0.01; ***p < 0.001; Student's *t* test. Data are mean ± SEM. ***K-M***, Linear correlation of spine density and percentage of stable (***K***), new (***L***), or eliminated (***M***) spines in DSCAM knockdown neurons. *R* = 0.7595 for stable spines, *R* = −0.4751 for new spines, and *R* = −0.6434 for eliminated spines.

Since DSCAM deficiency results in increased spine maturation, we investigated whether DSCAM modulates spine dynamics by knocking down DSCAM in neurons via small hairpin RNA (sh-RNA). Based on a previous report (Ly et al., 2008), we constructed sh-RNA for mouse or rat DSCAM (sh-DSCAM) and scrambled sh-RNA for use as a control (sh-control). We then confirmed the knockdown efficiency of sh-DSCAM in HEK293T cells transfected with mouse DSCAM-His (Fig. 6*A*). Furthermore, we observed and analyzed dendritic spine density in sh-DSCAM-transfected neurons. Compared with that in neurons transfected with sh-control, the total spine density was increased in neurons transfected with sh-DSCAM (Fig. 6*B*,*C*). To exclude the off-target effects of sh-DSCAM, we overexpressed human DSCAM (hDSCAM), which cannot be recognized by sh-DSCAM (Fig. 6*A*), in sh-DSCAM transfected neurons, and we found that hDSCAM overexpression rescued the spine deficits in DSCAM knockdown neurons (Fig. 6*B*,*C*), thus confirming the effects of DSCAM knockdown on spine development. Furthermore, the neurons were stained with anti-PSD-95 antibody to quantify the number and size of PSD-95 puncta. Compared with nontransfected neurons in the same coverslip or sh-control transfected neurons, neurons transfected with sh-DSCAM displayed increased numbers and sizes of PSD-95 puncta (Fig. 6*D–F*). These results indicate that knockdown of DSCAM in cultured neurons increases dendritic spine maturation.

Because enhanced spine formation or stabilization leads to increased spine maturation, we performed time-lapse imaging in cultured neurons to observe the dynamic spine maturation process. Cultured cortical neurons were cotransfected with GFP and sh-DSCAM or sh-control at DIV9 and imaged at DIV15-DIV17. The same secondary dendritic branch was imaged every minute for 1 h, and the percentages of stable, new, and eliminated spines were analyzed. As shown in Figure 6*G–J*, neurons transfected with sh-DSCAM exhibited an increase in stable spines and a decrease in eliminated spines, but they showed no change in the number of new spines during the imaging period compared with neurons transfected with sh-control. In other words, spines were more often stabilized and less often eliminated because of the knockdown of DSCAM. Notably, there was a positive correlation between the percentage of stable spines and spine density and a negative correlation between the percentage of eliminated spines and spine density (Fig. 6*K–M*). Together, these live-imaging data demonstrated that DSCAM knockdown promotes spine stabilization in neurons, thus accelerating spine maturation.

### DSCAM interacts with NLGN1

**Figure 7.**
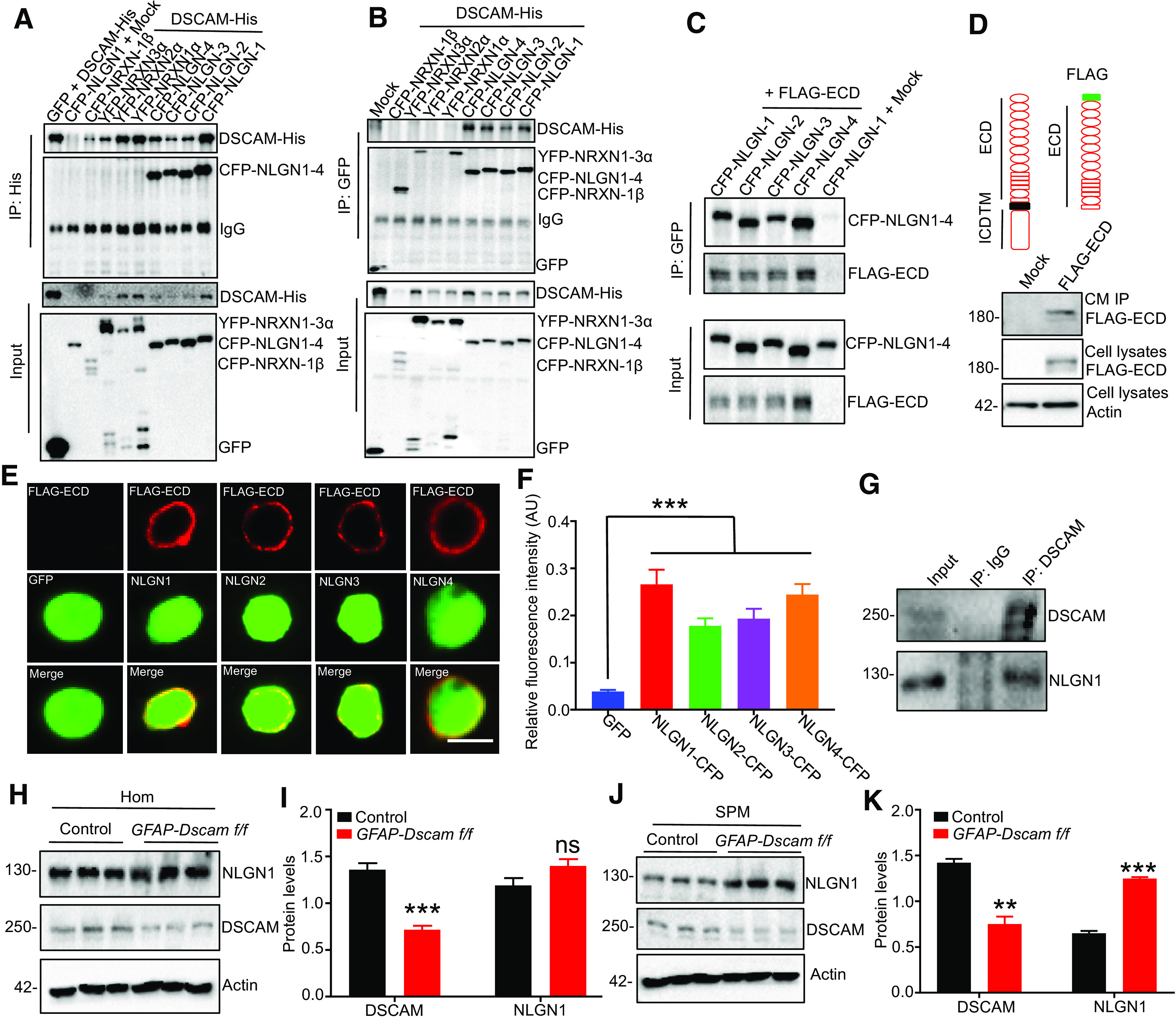
Interaction of DSCAM to NLGN1 through its ECD. ***A***, ***B***, DSCAM interacts with NLGNs. HEK293T cells were cotransfected with DSCAM-His and indicated constructs for coimmunoprecipitation assays with anti-His (***A***) or anti-GFP antibody (***B***). The inputs and resulting coimmunoprecipitation complexes were blotted with anti-GFP and anti-His antibodies. ***C***, DSCAM-ECD interacts with NLGNs. HEK293T cells were cotransfected with FLAG-tagged ECD of DSCAM (FLAG-ECD) or empty control (Mock) and indicated constructs for coimmunoprecipitation assay with anti-GFP antibody. ***D***, FLAG-ECD was secreted to the medium. HEK293T cells were transfected with FLAG-ECD or empty control. After 24 h, the medium was changed to CM containing 0.5% FBS for another 24 h. The CM was collected to immunoprecipitation with anti-FLAG antibody to enrich secretable FLAG-ECD. The resulting immunoprecipitation complexes and cell lysates were probed with anti-FLAG antibody. ***E***, ***F***, FLAG-ECD binds to the cell surface of cells expressing NLGNs. HEK293T cells were cotransfected with NLGN1-NLGN4 or empty vector plus GFP for 24 h and fixed. Before the immunostaining with anti-FLAG antibody, the fixed cells were incubated with CM containing FLAG-ECD for 4 h. Representative immunostaining images of cells treated with FLAG-ECD (***E***). Scale bar, 8 μm. Quantitative analysis of relative red fluorescence intensity (intensity of red fluorescence/green fluorescence) in ***E*** (***F***). Data were from three independent experiments and shown as mean ± SEM. ***p < 0.001 (one-way ANOVA). ***G***, Endogenous DSCAM interacts with NLGN1 *in vivo*. WT brain lysates were immunoprecipitated with anti-DSCAM antibody, and resulting complexes were blotted with anti-DSCAM and anti-NLGN1 antibodies. ***H***, ***I***, NLGN1 protein level was not altered in the *GFAP-Dscam f/f* mice. Whole-brain homogenates from *GFAP-Dscam f/f* and control mice were subjected to Western blotting with indicated antibodies (***H***). Quantitative analysis of data in ***H*** (***I***). *N* = 3 mice for each genotype. Data are mean ± SEM. ***p < 0.001 (two-way ANOVA). ***J***, ***K***, NLGN1 protein level was increased in SPM fraction of *GFAP-Dscam f/f* mice. SPM fractions from each genotype were probed with anti-DSCAM and anti-NLGN1 antibodies (***J***). Quantitative analysis of data in ***J*** (***K***). *N* = 9 mice for each genotype. Data are mean ± SEM. **p < 0.01; ***p < 0.001; two-way ANOVA.

DSCAM, a single transmembrane protein, belongs to the Ig superfamily of CAMs that plays a critical role in synapse formation and maturation (Sudhof, 2008, 2017). To explore the underlying mechanisms of DSCAM in spine maturation, we examined whether DSCAM interacts with other CAMs. NLGNs, a class of transmembrane proteins that interact *in trans* with presynaptic NRXNs, are classic postsynaptic CAMs and are essential for synaptogenesis and synapse maturation (Varoqueaux et al., 2006; Chubykin et al., 2007; Sudhof, 2008, 2017; Bemben et al., 2015). We cotransfected DSCAM-His with NLGNs (NLGN1-NLGN4) or NRXNs (NRXN1α-3α and 1β) into HEK293T cells and performed coimmunoprecipitation assays. As shown in Figure 7*A*, NLGN1-NLGN4 were coimmunoprecipitated with DSCAM-His, but NRXNs were not. Conversely, DSCAM-His was also coimmunoprecipitated with NLGNs (Fig. 7*B*). Moreover, the FLAG-tagged ECD of DSCAM (FLAG-ECD) and NLGNs was cotransfected into HEK293T cells for coimmunoprecipitation assay, and FLAG-ECD was coimmunoprecipitated with NLGNs (Fig. 7*C*). These results suggest that DSCAM could interact with NLGN1-NLGN4 through its ECD. To detect whether DSCAM-ECD binds to cell surface NLGNs, we performed a cell surface binding assay. First, we overexpressed FLAG-ECD in HEK293T cells. To confirm whether FLAG-ECD could be secreted into the medium, we collected conditional medium (CM) and added anti-FLAG antibody to enrich the secretable FLAG-ECD. As shown in Figure 7*D*, FLAG-ECD was detected in IPed CM, as well as in cell lysates. Next, we harvested CM with FLAG-ECD or control and added them to fixed HEK293T cells cotransfected with NLGNs and GFP. After incubation, the cells were subjected to immunostaining with anti-FLAG antibody. As shown in Figure 7*E*, FLAG-ECD bound to the surface of cells transfected with NLGNs. Indeed, more FLAG-ECD was enriched on the cell surface that expressed NLGN1 than on the cell surfaces that expressed NLGN2-NLGN4 (Fig. 7*F*). Together, these results suggest that DSCAM interacts with NLGN1 through their ECDs.

NLGN1 is strongly expressed in excitatory synapses and is implicated in ASDs (Varoqueaux et al., 2006; Sudhof, 2008, 2017; Nakanishi et al., 2017). Next, we focused on the DSCAM-NLGN1 interaction. The endogenous interaction of DSCAM and NLGN1 was confirmed by coimmunoprecipitation with mouse brain lysates (Fig. 7*G*). We then examined whether DSCAM can regulate NLGN1 expression and distribution in the synaptic area. The protein level of NLGN1 was comparable in the brain between control and DSCAM-deficient mice (Fig. 7*H*,*I*). However, the level of NLGN1 in the SPM fraction was increased in *GFAP-Dscam f/f* mice (Fig. 7*J*,*K*), suggesting that DSCAM deficiency can induce NLGN1 enrichment in the synaptic membrane.

### DSCAM inhibits the NLGN1-NRXN1β interaction

**Figure 8.**
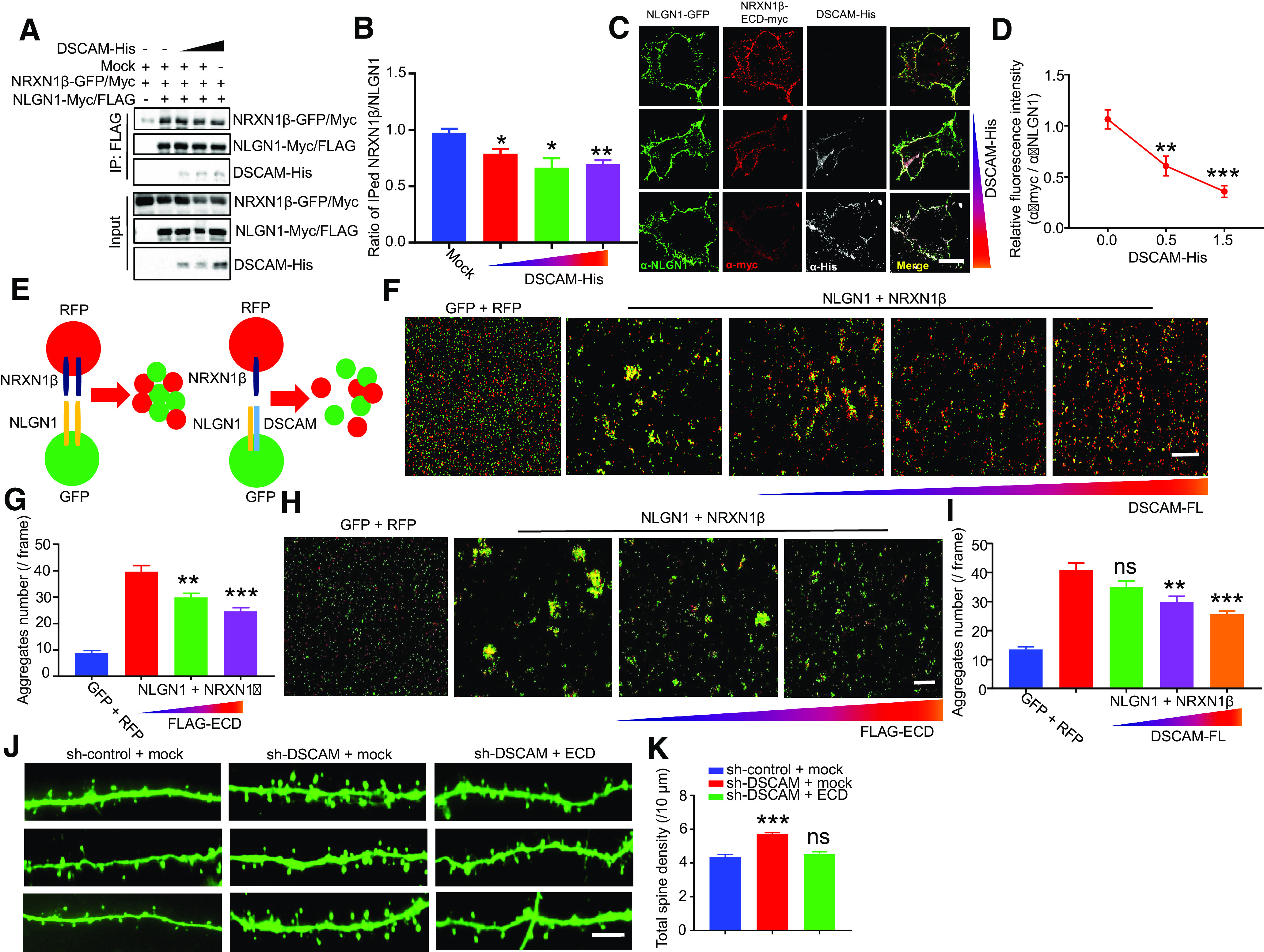
Inhibition of NLGN1-NRXN1β interaction by DSCAM. ***A***, ***B***, DSCAM inhibits the NLGN1-NRXN1β interaction. HEK293T cells were cotransfected with different amounts of DSCAM-His and indicated constructs. Cell lysates were IPed with anti-FLAG antibody, and resulting complexes were blotted with anti-GFP, anti-FLAG, and anti-His antibodies (***A***). The relative intensity of coimmunoprecipitated NRXN1β (intensity of coimmunoprecipitated NRXN1β/IPed NLGN1) was quantified in ***B***. Data were from three independent experiments and shown as mean ± SEM. *p < 0.05; **p < 0.01; one-way ANOVA. ***C-G***, DSCAM inhibits transcellular NLGN1-NRXN1β interaction in cell surface binding assay (***C***,***D***) and cell aggregation assay (***E-G***). COS-7 cells were cotransfected with NLGN1 and increasing amounts of DSCAM-His for 24 h and incubated with purified NRXN1β-ECD-myc for 1 h before fixation. Fixed cells were immunostained with anti-NLGN1, anti-myc, and anti-His antibodies. Representative immunostaining images of cells (***C***). Scale bar, 10 μm. Quantitative analysis of relative red fluorescence intensity (intensity of red fluorescence/green fluorescence) in ***C*** (***D***). Data were from three independent experiments and shown as mean ± SEM. **p < 0.01; ***p < 0.001; one-way ANOVA. Schematic illustration of cell aggregation assay (***E***). HEK293T cells expressing NRXN1β or empty vector with RFP (red cells) were mixed with cells expressing NLGN1 and GFP plus different amounts of DSCAM (green cells). After 1 h, cell mixtures were imaged (***F***) and cell aggregates were counted (***G***). Scale bar, 200 μm. *N* = 15 images for each condition from three independent experiments. Data are mean ± SEM. **p < 0.01; ***p < 0.001; one-way ANOVA. ***H***, ***I***, DSCAM-ECD disrupts transcellular NLGN1-NRXN1β interaction. Different amounts of CM with FLAG-ECD were added into HEK293T cell mixtures of NRXN1β (red cells) and NLGN1 (green cells). Cell aggregates were imaged (***H***) and counted (***I***). Scale bar, 200 μm. Data were from three independent experiments and shown as mean ± SEM. **p < 0.01; ***p < 0.001; one-way ANOVA. ***J***, ***K***, DSCAM-ECD rescued spine overmaturation in DSCAM knockdown neurons. Primary cortical neurons were transfected at DIV9 with sh-DSCAM or sh-control. At DIV15, transfected neurons were incubated with CM containing FLAG-ECD or mock for 2 d and fixed. Representative images of dendritic spines (***J***). Scale bar, 10 μm. Quantitative analysis of total spine density in ***J*** (***K***). *N* = 20 neurons for sh-control + mock; *n* = 48 neurons for sh-DSCAM + mock; *n* = 21 neurons for sh-DSCAM + FLAG-ECD. Data are mean ± SEM. **p < 0.01; ***p < 0.001; one-way ANOVA.

Postsynaptic NLGN1 is involved in synapse maturation by interacting *in trans* with presynaptic NRXN1β (Sudhof, 2008). We examined whether DSCAM could regulate the NLGN1-NRXN1β interaction by cotransfecting NLGN1, NRXN1β, and DSCAM into HEK293T cells. As shown in Figure 8*A*, *B*, NRXN1β was coimmunoprecipitated with NLGN1 in the absence of DSCAM-His. However, NRXN1β coimmunoprecipitation with NLGN1 was decreased in the presence of increased DSCAM-His, suggesting that DSCAM inhibits the NLGN1-NRXN1β interaction. Since NLGN1 binds *in trans* with NRXN1β, we performed cell surface binding assays to detect whether DSCAM could inhibit this trans-cell interaction. COS-7 cells were cotransfected with NLGN1 and varied amounts of DSCAM-His. After transfection, the cells were treated with purified NRXN1β-ECD-myc protein for 1 h and then fixed for immunostaining with anti-NLGN1, anti-myc, and anti-His antibodies. As shown in Figure 8*C*, *D*, the red fluorescence, which indicates cell surface binding of NRXN1β-ECD-myc, was decreased when DSCAM-His expression was increased. Moreover, we also used cell aggregation assays to confirm the inhibition of NLGN1-NRXN1β trans-cell interactions by DSCAM (Fig. 8*E*). Cells clustered when HEK293T cells cotransfected with NLGN1 and GFP were mixed with another set of HEK293T cells cotransfected with NRXN1β and RFP (Fig. 8*F*). DSCAM-His coexpression with NLGN1 in GFP^+^ cells decreased the number of aggregates in a dose-dependent manner (Fig. 8*F*,*G*). Together, these results suggest that DSCAM inhibits the NLGN1-NRXN1β trans-cell interaction.

As DSCAM inhibited the NLGN1-NRXN1β trans-cell interaction through ECD binding to NLGN1, we contemplated whether DSCAM-ECD alone could block the NLGN1-NRXN1β *in trans* interaction. Using cell aggregation assays, we added CM containing FLAG-ECD to mixtures of HEK293T cells expressing either NLGN1 or NRXN1β. As shown in Figure 8*H*, *I*, DSCAM-ECD reduced the number of NLGN1-NRXN1β cell aggregates in a dose-dependent manner. Next, we theorized that DSCAM-ECD could rescue spine overmaturation caused by DSCAM deficiency. We treated DSCAM knockdown neurons with CM containing FLAG-ECD and found that FLAG-ECD rescued the increased total spine densities induced by DSCAM knockdown (Fig. 8*J*,*K*). Together, these results suggest that DSCAM may block NLGN1-NRXN1β interactions to inhibit premature spine maturation.

### DSCAM deficiency induces autism-like behaviors and enhances spatial memory in mice

**Figure 9.**
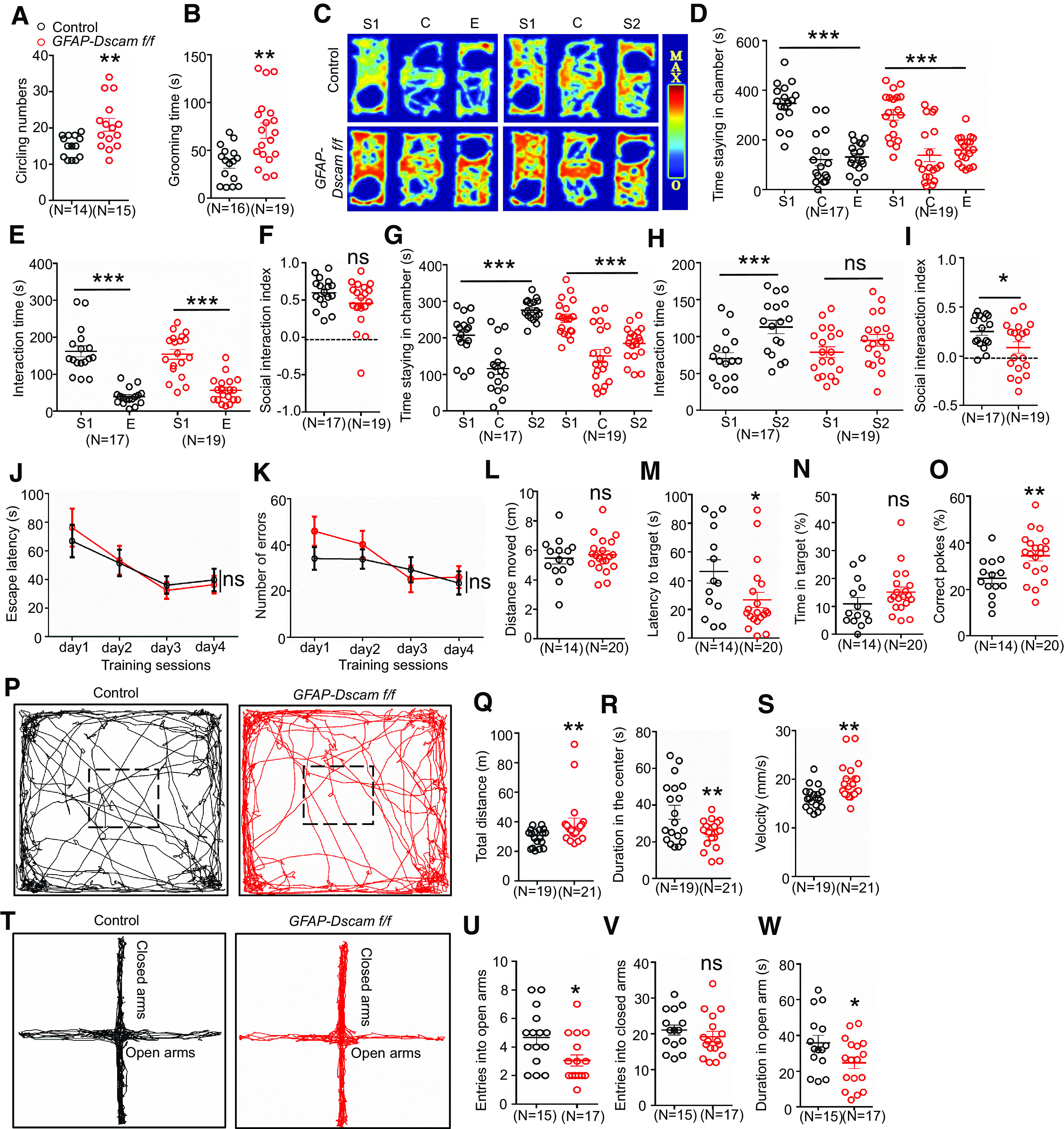
Autism-like behaviors and enhanced spatial memory in *GFAP-Dscam f/f* mice. ***A***, ***B***, *GFAP-Dscam f/f* mice displayed increased circling and grooming in OFT. During 10 min OFT, mice circling numbers (***A***) and grooming time (***B***) were counted. For circling, *n* = 14 for control mice, *n* = 15 for *GFAP-Dscam f/f* mice; for grooming, *n* = 16 for control mice, *n* = 19 for *GFAP-Dscam f/f* mice. Data are mean ± SEM. **p < 0.01 (Student's *t* test). ***C-I***, *GFAP-Dscam f/f* mice displayed reduced social novelty. Representative heat map for the mice movements in three-chamber social interaction test (C). S1, Stranger1 mouse; S2, stranger2 mouse; E, empty cage; C, center chamber. Quantitative analysis of time staying in each chamber (***D***,***G***), interaction time (***E***,***H***), and social interaction index (***F***,***I***). Social interaction index was calculated as follows: in ***F***, [interaction time (S1) – interaction time (***E***)]/[interaction time (S1) + interaction time (***E***)] for each genotype; in ***I***, [interaction time (S2) – interaction time (S1)]/[interaction time (S2) + interaction time (S1)] for each genotype. *N* = 17 mice for control, *n* = 19 for *GFAP-Dscam f/f* mice. Data are mean ± SEM. NS, *p* > 0.05. ***p < 0.001; Student's *t* test for ***F*** and ***I***, two-way ANOVA for ***D***, ***E***, ***G***, and ***H***. ***J***, ***K***, *GFAP-Dscam f/f* mice displayed elevated spatial memory in Barnes maze. Quantitative analysis of escape latency (***J***) and error numbers (***K***) in a 4 d training session. Quantitative analysis of moved distances (***L***), latency to target (***M***), time in target (***N***), and correct pokes (***O***) in the test session. *N* = 14 for control mice, *n* = 20 for *GFAP-Dscam f/f* mice. Data are mean ± SEM. NS, *p* > 0.05. *p < 0.05; **p < 0.01; Student's *t* test for ***L*** and ***O***, two-way ANOVA for ***J*** and ***K***. ***P-S***, *GFAP-Dscam f/f* mice displayed anxiety-like behavior in OFT. Male 2-month-old mice were placed in the chambers, and movement was monitored for 10 min. Representative traces of mice in OFT (***P***). Square with a dotted line represents the center area. Quantitative analysis of traveled distance (***Q***), duration in the center (***R***), and velocity (***S***). *N* = 19 for control mice, *n* = 21 for *GFAP-Dscam f/f* mice. Data are mean ± SEM. **p < 0.01, Student's *t* test for ***R*** and ***S***, Mann–Whitney *U* test for ***Q***. ***T-W***, *GFAP-Dscam f/f* mice displayed anxiety-like behavior in EPM. Mice were put in the center of the EPM, and they could freely explore for 10 min. The time stayed in and entries into the open arms were recorded. Representative traces of mice in EPM (***T***). Quantitative analysis of entries into the open arms (***U***) and the closed arms (***V***), as well as duration in the open arms (***W***). *N* = 15 for control mice, *n* = 17 for *GFAP-Dscam f/f* mice. Data are mean ± SEM. *p < 0.05 (Student's *t* test).

Given that *DSCAM* is 1 of 102 ASD predominant genes and that its absence accelerated spine maturation and elevated glutamatergic transmission, we next examined the behaviors of DSCAM-deficient mice. First, we conducted autism-associated behavioral tests in *GFAP-Dscam f/f* and control mice. As shown in Figure 9*A*, *B*, *GFAP-Dscam f/f* mice displayed increased amounts of circling and self-grooming than control mice did.

Along with repetitive behaviors, profound social impairment is a core symptom of ASD. We therefore conducted three-chamber social interaction tests (Fig. 9*C–I*) for social recognition and social interaction (Moy et al., 2004). In the social preference test, both *GFAP-Dscam f/f* and control mice preferred to explore a stranger mouse (stranger1 mouse [S1]) than an inanimate object (empty cage [E]), as measured by duration spent in each chamber and duration of sniffing (interaction time) (Fig. 9*C–E*). Moreover, the social interaction index was indistinguishable between the two genotypes, suggesting normal social preference in *GFAP-Dscam f/f* mice (Fig. 9*F*). Next, we compared social novelty preference. After the E was replaced with another stranger mouse (stranger2 mouse [S2]), control mice spent more time in the S2 chamber and interacted more with S2 than they did with the familiar mouse (S1) (Fig. 9*G*,*H*). In contrast, *GFAP-Dscam f/f* mice spent less time in the S2 chamber than in the S1 chamber (Fig. 9*G*). Additionally, *GFAP-Dscam f/f* mice did not differentiate between S1 and S2 in terms of sniffing duration (Fig. 9*H*). Social novelty index score was decreased in *GFAP-Dscam f/f* mice (Fig. 9*I*), suggesting an impairment in social novelty but not sociability.

We also conducted the Barnes circular mazes test to examine the impact of DSCAM deficiency on spatial learning and memory (Fig. 9*J–O*). In the 4 d training session, escape latency and number of errors were similar in both genotype (Fig. 9*J*,*K*), suggesting normal spatial learning for *GFAP-Dscam f/f* mice. However, at test, *GFAP-Dscam f/f* mice displayed reduced latency to reach target and greater numbers of correct pokes, but they showed no difference in travel distance and time on target, compared with the controls (Fig. 9*L–O*), which suggests that DSCAM deletion may enhance spatial memory. Together, the data indicate that *GFAP*-mediated DSCAM deficiency increases stereotyped behaviors, impairs social novelty, and enhances spatial memory.

Moreover, *GFAP-Dscam f/f* mice exhibited increased distance traveled and velocity in the OFT but decreased time in the center (Fig. 9*P–S*). In the EPM, *GFAP-Dscam f/f* mice entered the open arms less and spent less time in them (Fig. 9*T–W*), suggesting increased anxiety-like behaviors because of DSCAM deletion in neurons and astrocytes.

### DSCAM deficiency in pyramidal neurons induces premature spine maturation and autism-like behaviors

**Figure 10.**
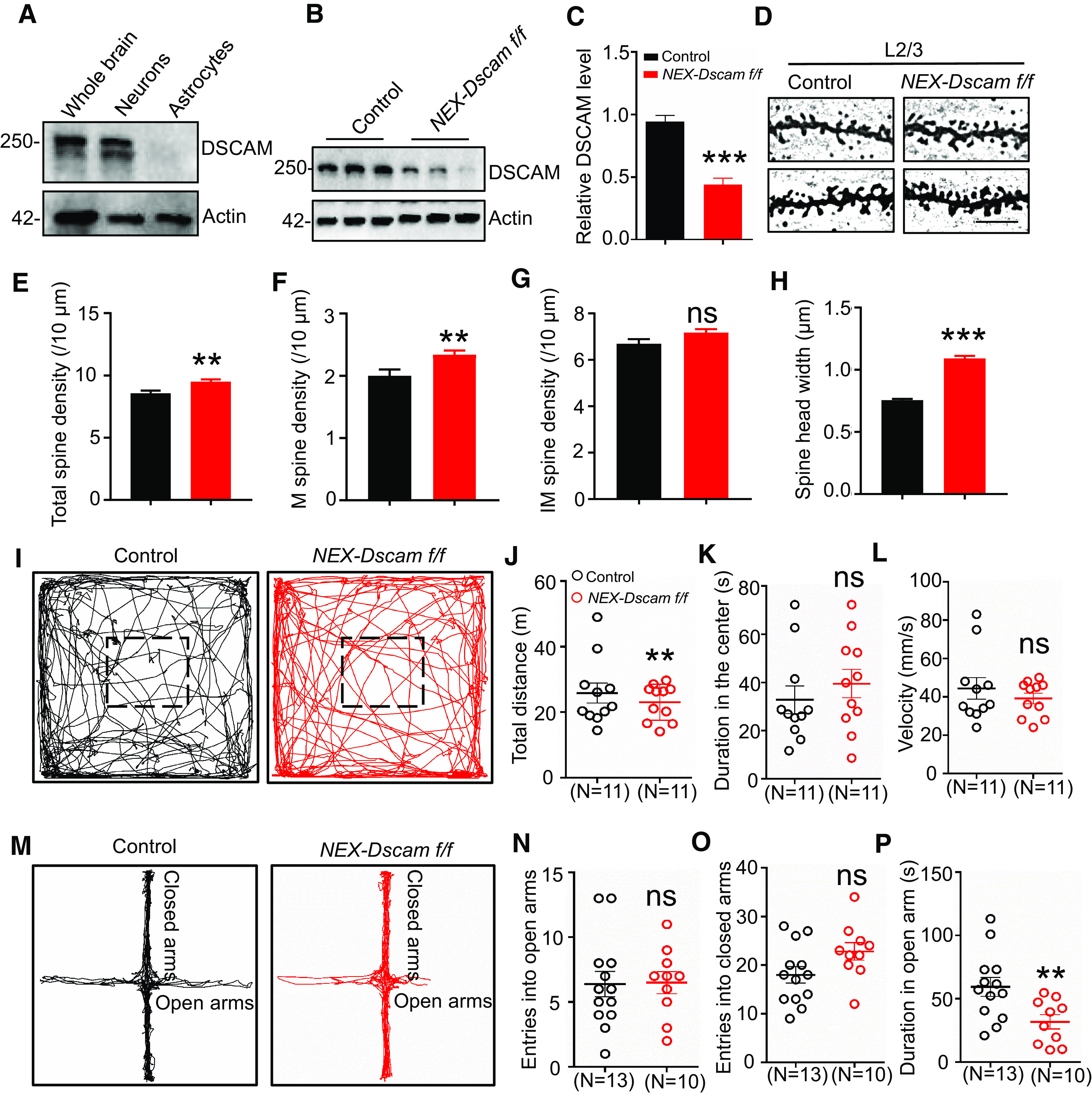
Spine overmaturation in *NEX-Dscam f/f* mice. ***A***, DSCAM is expressed in neurons. Whole-brain lysates, as well as cell lysates of cultured neurons and astrocytes from mice were probed with anti-DSCAM antibody. ***B***, ***C***, DSCAM expression is reduced in *NEX-Dscam f/f* mice. Lysates of isolated sensory cortex from each genotype were probed with anti-DSCAM antibody (***B***). Quantitative analysis of relative DSCAM intensity in ***B*** (***C***). *N* = 3 mice for each genotype. Data are mean ± SEM. **p < 0.01 (Student's *t* test). ***D-H***, *NEX-Dscam f/f* mice displayed increased spine maturation in the sensory cortex L2/3. Representative Golgi staining images of dendritic spines in sensory cortex (***D***). Scale bar, 10 μm. Quantitative analysis of total (***E***), M (***F***), and IM (***G***) spine density, as well as spine head width (***H***). *N* = 3 mice for each genotype. Data are mean ± SEM. *p < 0.05; **p < 0.01; ***p < 0.001; Student's *t* test. ***I-L***, *NEX-Dscam f/f* mice displayed normal in OFT. Representative traces of mice in OFT (***I***). Quantitative analysis of traveled distances (***J***), duration in the center (***K***), and velocity (***L***). *N* = 11 for control mice, *n* = 11 for *NEX-Dscam f/f* mice. Data are mean ± SEM. NS, *p* > 0.05. **p < 0.01; Student's *t* test for ***K*** and ***L***, Mann–Whitney *U* test for ***J***. ***M-P***, *NEX-Dscam f/f* mice displayed normal behaviors in EPM. Representative traces of mice in EPM (***M***). Quantitative analysis of entries into the open arms (***N***) and the closed arms (***O***), as well as duration in the open arms (***P***). *N* = 13 for control mice, *n* = 10 for *NEX-Dscam f/f* mice. Data are mean ± SEM. NS, *p* > 0.05. **p < 0.01 (Student's *t* test).

DSCAM was primarily expressed in neurons, as examined by Western blotting with isolated neurons and astrocytes (Fig. 10*A*), and its expression in cultured neurons was negatively regulated during the postnatal development period (Fig. 1*C*,*D*). We therefore investigated whether the spine and behavioral deficits in *GFAP-Dscam f/f* mice could result from DSCAM deficiency in pyramidal neurons. To this end, we crossed *Dscam f/f* mice with a *NEX*-Cre line, in which Cre is expressed in pyramidal neurons of the neocortex and hippocampus. We found that the DSCAM level was reduced to 50% in the cortex of the resultant *NEX-Dscam f/f* mice (Fig. 10*B*,*C*). Furthermore, the sensory cortex L2/3 of *NEX-Dscam f/f* mice at P42 was subjected to Golgi staining. The densities of total spines and mature spines were markedly increased, whereas the density of immature spines remained unchanged (Fig. 10*D–G*). Additionally, the width of spine head increased (Fig. 10*H*). These results suggest that DSCAM deficiency in pyramidal neurons also led to spine overmaturation.

Next, we examined the behaviors of *NEX-Dscam f/f* mice. In the OFT, the duration spent in the center and the velocity were similar between *NEX-Dscam f/f* and control mice (Fig. 10*I–L*). In the EPM, the *NEX-Dscam f/f* mice entered open and closed arms at similar rates as the control mice did, but they reduced the duration spent in open arms (Fig. 10*M–P*), suggesting that DSCAM deficiency in pyramidal neurons does not induce anxiety-like behaviors.

**Figure 11.**
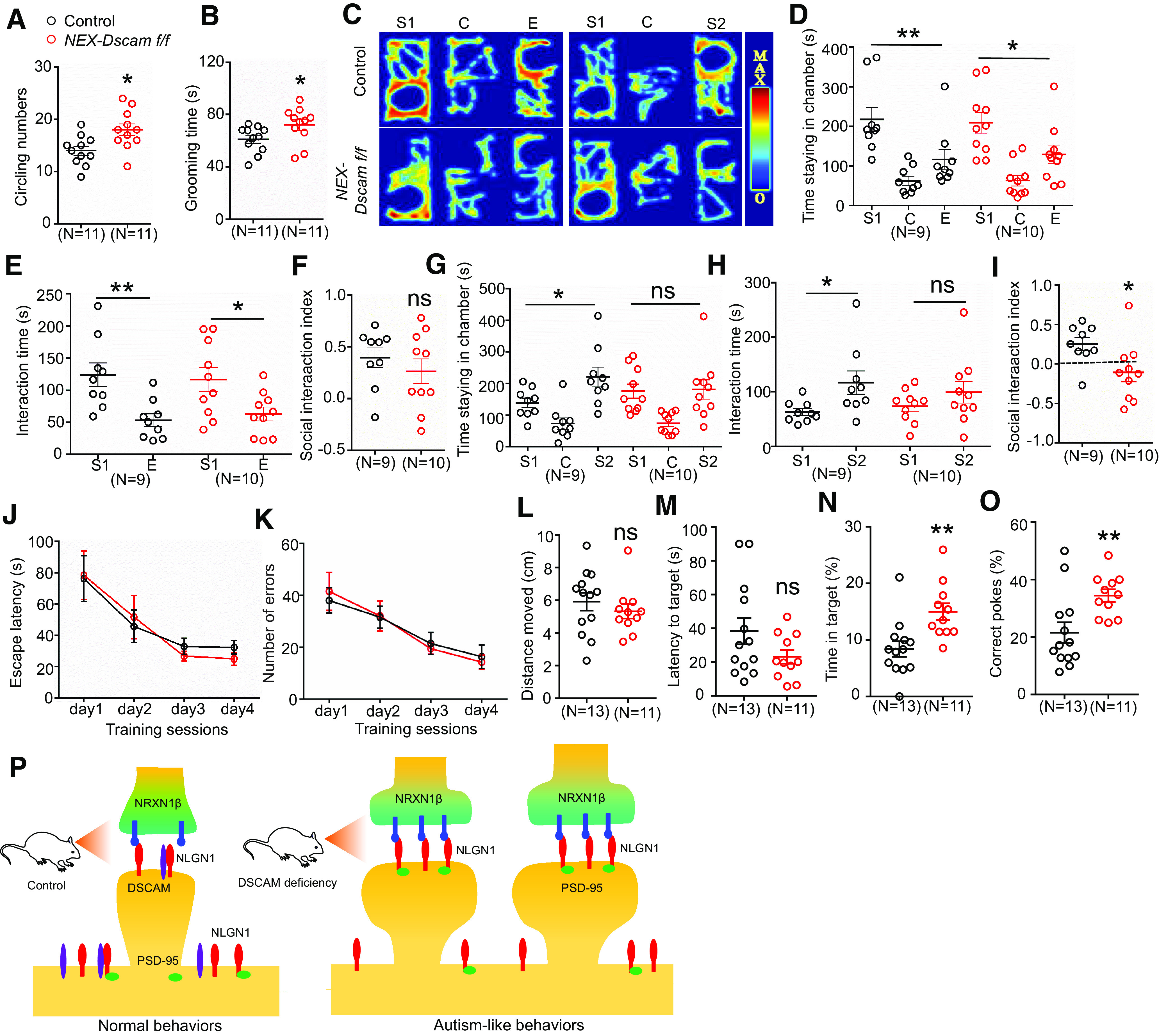
Autism-like behaviors and enhanced spatial memory in *NEX-Dscam f/f* mice. ***A***, ***B***, *NEX-Dscam f/f* mice displayed increased circling and grooming in OFT. During 10 min OFT, mice circling numbers (***A***) and grooming time (***B***) were counted. *N* = 11 for control mice, *n* = 11 for *NEX-Dscam f/f* mice. Data are mean ± SEM. *p < 0.05 (Student's *t* test). ***C-I***, *NEX-Dscam f/f* mice displayed reduced social novelty. Representative heat map for the mice movements in three-chamber social interaction test (***C***). Quantitative analysis of time staying in each chamber (***D***,***G***), interaction time (***E***,***H***), and social interaction index (***F***,***I***). *N* = 9 mice for control, *n* = 10 for *NEX-Dscam f/f* mice. Data are mean ± SEM. NS, *p* > 0.05. *p < 0.05; **p < 0.01; Student's *t* test for ***E***, ***F***, ***H***, and ***I***; one-way ANOVA for *NEX-Dscam f/f* group in ***D*** and control group in ***G***; Kruskal–Wallis test for control group in ***D*** and *NEX-Dscam f/f* group in ***G***. ***J-O***, *NEX-Dscam f/f* mice displayed elevated spatial memory in Barnes maze. Quantitative analysis of escape latency (***J***) and error numbers (***K***) in 4 d training session. Quantitative analysis of moved distances (***L***), latency to target (***M***), time in target (***N***), and correct pokes (***O***) in the test session. *N* = 13 for control mice, *n* = 11 for *NEX-Dscam f/f* mice. Data are mean ± SEM. NS, *p* > 0.05. *p < 0.05; **p < 0.01; two-way ANOVA for ***J***, ***K***; Student's *t* test for ***L***, ***M***, and ***O***; Mann–Whitney *U* test for ***N***. ***P***, Schematic illustration for DSCAM deficiency on spine maturation.

In addition, *NEX-Dscam f/f* mice were subjected to tests for autism-associated behaviors. *NEX-Dscam f/f* mice exhibited stereotyped behaviors (Fig. 11*A*,*B*), social novelty deficits (Fig. 11*C–I*), and enhanced spatial memory (Fig. 11*J–O*). Together, these results demonstrate that DSCAM deficiency in pyramidal neurons also induces autism-like behaviors and enhanced spatial memory. Therefore, we propose our DSCAM model of synapse maturation. In control mice, DSCAM was localized to immature spines and interacted with NLGN1 to inhibit the NLGN1-NRXN1β interaction, thus preventing spine overmaturation. In DSCAM-deficient mice, this inhibition of the NLGN1-NRXN1β interaction was removed, and resulted in premature spine maturation, which ultimately led to autism-like behaviors and enhanced spatial memory (Fig. 11*P*).

## Discussion

In this study, we provided evidence that DSCAM serves as a repressor for the NLGN1-NRXN1β interaction, and that its deficiency leads to premature spine maturation and excessive glutamatergic transmission, inducing autism-like behaviors. The major findings of this paper are as follows. First, DSCAM was downregulated following synapse maturation, and knockdown of DSCAM in primary neurons increased spine maturation. Second, *in vivo*, *GFAP*-mediated DSCAM deficiency in mice led to spine overmaturation and increased glutamatergic transmission. Third, DSCAM interacted with NLGN1 through its ECD and blocked the NLGN1-NRXN1β interaction to suppress spine overmaturation. Finally, DSCAM-deficient mice exhibited autism-like behaviors, including impaired social novelty, increased circling and grooming time, and improved spatial memory.

Dendritic spines are small protrusions from the dendrites of excitatory neurons and play critical roles in learning, memory, and cognition (Yuste and Bonhoeffer, 2004; Berry and Nedivi, 2017). Dysregulation of spine development has been implicated in mental disorders, including autism, depression, and schizophrenia (Forrest et al., 2018). Previous studies have shown that DSCAM acts as a crucial factor or regulator in synapse development and synaptic plasticity (Yamagata and Sanes, 2008; H. L. Li et al., 2009; Maynard and Stein, 2012; Thiry et al., 2016; Laflamme et al., 2019), indicating its role in spine development. Here, we found an increase in the density of matured spines and in the volume of spine heads in *GFAP-Dscam f/f* and *NEX-Dscam f/f* mice. Because of potential differences in staining efficiency between the two batches of Golgi staining, we observed a small difference in the spine density data for the control mice between Figures 3*B–D* and 11*E–G*. We further confirmed the spine deficits in cultured neurons. Live-imaging results showed that increased spine density could be attributed to enhanced stability. These results suggest that DSCAM can downregulate spine maturation in the developing sensory cortex.

Several recent studies reported that DSCAM-ICD plays a critical role in synapse development and neuronal migration (Sachse et al., 2019; Arimura et al., 2020). However, we found that DSCAM interacted with NLGN1 through its ECD to disrupt the NLGN1-NRXN1β interaction. NLGN1 plays an essential role in spine maturation (Varoqueaux et al., 2006; Chubykin et al., 2007; Bemben et al., 2015). DSCAM-ECD also rescued abnormal spine maturation in DSCAM knockdown neurons. Therefore, DSCAM might modulate spine maturation through its ECD. Differences in spine densities have previously been reported in patients with neuropsychiatric disorders, such as increased spine densities in L2 of the cortex and L5 of the temporal lobe in ASD patients (Hutsler and Zhang, 2010). In addition, increased spines were also observed in Fragile-X syndrome patients and corresponding mouse models (Comery et al., 1997; Irwin et al., 2000). Thus, dendritic spine malformation may lead to neuronal dysfunction during neural development, and DSCAM may serve as a modulator in spine remodeling.

Disruption of the excitatory/inhibitory (E/I) balance has been implicated in various neuropsychiatric disorders, including ASD (Rubenstein and Merzenich, 2003; Nelson and Valakh, 2015). Elevation of the E/I ratio in the mouse PFC leads to social deficits (Yizhar et al., 2011). In a similar manner, we found that glutamatergic transmission increased in *GFAP-Dscam f/f* mice during postnatal development while GABAergic transmission was unchanged, which might result in elevation of the E/I ratio and lead to social novelty deficits (Nelson and Valakh, 2015). Social interaction deficits were found in these DSCAM-deficient mice. Although some ASD mouse models exhibit a reduced E/I ratio, such as mice with autism-associated mutations in NRXNs or SHANKs (Etherton et al., 2009; Jiang and Ehlers, 2013), an enhanced E/I ratio can also be observed in many other ASD mouse models, such as TSC1-, MDGA2-, FMRP-, or CUL3-deficient mice (Bateup et al., 2013; Zhang et al., 2014; Connor et al., 2016; Dong et al., 2020). Precocious spine maturation in hippocampal neurons dramatically elevated the E/I ratio and impaired the cognitive development in SynGAP mutant mice (Clement et al., 2012). In our autistic model of DSCAM-deficient mice, early spine maturation in DSCAM-deficient neurons contributed to increased excitatory synaptic transmission, which might impair the E/I balance and lead to autism-like behaviors. In addition, DSCAM deficiency increased only mEPSC frequency, not amplitude, which may account for the normal surface distribution of active AMPARs or NMDARs.

Previous studies have indicated higher cognitive function in patients with macrocephaly and ASD (Courchesne and Pierce, 2005; Sacco et al., 2007). Increased head circumferences have been shown in ASD patients with special capabilities (Bena et al., 2013). *Dscam*-null mice (DSCAM^del17^ or DSCAM^2J^) similarly exhibited ventricular dilation with a dome-shaped head (Xu et al., 2011; Lemieux et al., 2016). Intriguingly, increased spatial memory was observed in both *GFAP-Dscam f/f* and *NEX-Dscam f/f* mice along with increased brain weight in this study, which implies that brain enlargement might be one factor explaining the enhanced spatial memory in DSCAM-deficient mice. Relatedly, it was previously theorized that mushroom spines (matured spines) can act as memory spines (Bourne and Harris, 2007). More mature spines might be another explanation for the increased spatial memory. Our live-imaging results found a positive correlation between the percentage of stable spines and the spine density, which may also contribute to the enhanced spatial memory demonstrated by DSCAM-deficient mice. It has also been reported that NLGN3-R451C mice (an ASD-associated mutation) displayed enhanced spatial learning in the Morris water maze in addition to autism-like behaviors (Tabuchi et al., 2007). Together, our results reveal the regulatory functions of DSCAM in spine maturation and its underlying molecular mechanisms in the nervous system. DSCAM-deficient mice with increased spatial memory might belong to the subset of autism mouse models with high functions.

*DSCAM* is located on chromosome 21 in humans; an extra copy of chromosome 21 causes Down syndrome (DS) (Saito et al., 2000). Upregulated DSCAM expression is found in the brains of DS patients, DS mouse models, and AD mouse models (Saito et al., 2000; Amano et al., 2004; Jia et al., 2011), causing pathologic changes in the brain and finally leading to impairment in cognitive functions. Elevated levels of DSCAM are also found in postmortem brains from bipolar subjects (Amano et al., 2008). Likewise, DSCAM expression is higher in intractable epilepsy patients than in controls (Shen et al., 2011). However, DSCAM deficiency has been found to disrupt dendritic self-avoidance and tiling in the mouse retina and cause abnormal neuronal migration (Fuerst et al., 2008, 2009; Sachse et al., 2019; Arimura et al., 2020). Additionally, premature termination mutations of DSCAM are observed in some ASD patients (Iossifov et al., 2014; T. Wang et al., 2016; Stessman et al., 2017; Yuen et al., 2017). Our results showed that loss of DSCAM in pyramidal neurons induced autism-like behaviors in mice. Together, all these findings suggest that dysregulated DSCAM expression alters spine maturation, resulting in abnormal synaptic transmission, which may explain how gene-dosage imbalance of DSCAM could potentially contribute to the pathogenesis of neurologic DSCAM-related disorders.
